# Efficiency comparison of DNA extraction kits for analysing the cockle gut bacteriome

**DOI:** 10.1016/j.heliyon.2024.e38846

**Published:** 2024-10-09

**Authors:** Catarina F. Lourenço, Ana R. Almeida, Amadeu M.V.M. Soares, Catarina R. Marques

**Affiliations:** Center for Environmental and Marine Studies (CESAM) & Department of Biology, University of Aveiro, Campus Universitário de Santiago, 3810-193, Aveiro, Portugal

**Keywords:** *Cerastoderma edule*, DNA extraction, DGGE, NGS, Bacterial community

## Abstract

Cockles play a vital ecological role and provide valuable ecosystem services globally. However, the performance, production, and safe consumption of cockles are significantly influenced by their gut-associated bacteriome. Accurate understanding of gut-bacteriome interactions, and surveillance of pathogenic bacteria loads in cockles, rely on efficient DNA extraction methods that yield high-quality and representative bacterial DNA. Despite this importance, reliable extraction methods for cockles are currently overlooked. Therefore, we evaluated the performance of five DNA extraction kits (E.Z.N.A.® Soil DNA; FastDNA® Spin; DNeasy PowerSoil Pro; QIAamp PowerFecal DNA; ZymoBIOMICS™DNA Miniprep) in terms of DNA quality, yield, bacterial community structure (analysed by using denaturating gradient gel electrophoresis; DGGE), and bacteriome composition (analysed by 16S rRNA gene sequencing) in *Cerastoderma edule* gut. The DNeasy kit provided the highest purity and quantity of bacterial DNA, while the PowerFecal and Zymo kits exhibited reduced extraction efficiency. DGGE profiles revealed significant variability between the tested kits (R = 0.512; mean P = 0.011), but the FastDNA kit under-represented the bacterial community in cockles’ gut. Based on alpha diversity, the DNeasy kit outperformed the others and successfully detected all abundant genera found with the alternative kits. Our findings indicate that the DNeasy kit is an efficient DNA extraction method, enabling a molecular representation of the gut-associated bacteriome in *C. edule*. These results contribute to the development of effective techniques for studying the cockle gut bacteriome and its ecological implications.

## Graphical abstract

**Figure:**
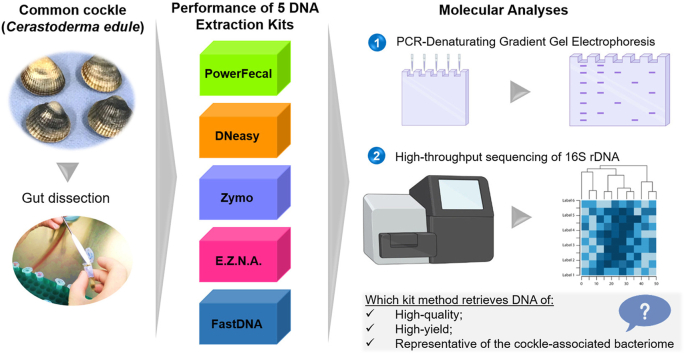

## 1 Introduction

Cockles are bivalves with a crucial role in several ecosystem functions, such as nutrient (re)cycling, water filtration, mitigation of habitat erosion, reduction of toxin and pathogen loads, support of benthic communities and pelagic food webs. Additionally, cockles deliver relevant ecosystem services, like shell-based materials for construction, agriculture and ornaments [1], and food production, given their high nutritional value [2,3]. Therefore, cockles are one of the most exploited bivalves in Europe, especially in Portugal, Spain, Ireland, France, and the United Kingdom, with nearly a total of 30k tons produced in 2017, and 25k tons in 2019 [[4], [5], [6]].

The filter-feeding behaviour of bivalves promotes the accumulation of beneficial and pathogenic bacteria in their tissues and gut [7]. As a result, bivalves can harbour a diverse gut bacteriome, which in turn modulates their immune system, physiological status and survival [8,9]. Therefore, it is of utmost relevance to understand dynamic shifts in the structure of gut bacteriome in response to environmental changes, pathogen-driven diseases, and other stressors that may impair bivalves' survival in ecosystems, as well as their production yields [10,11]. Moreover, since bivalves, including cockles, are a food resource usually consumed raw or slightly cooked, the presence of pathogenic bacteria must be regularly monitored to avoid foodborne diseases that jeopardise public health [7,9,12]. To this end, there is a method legally established at the European level that is based on the enumeration of *Escherichia coli* (standard: ISO 16649–3:2015), which is a pathogen (belonging to the Enterobacteriaceae family) often abundant in bivalves produced in areas highly exposed to faecal contamination [13]. Notwithstanding, cockles can accumulate other pathogenic bacteria than *E. coli* (*e.g.*, *Vibrio* spp.), whose bioloads cannot be reduced to levels safe for human consumption, even after subjecting bivalves to disinfection and sanitization processes (*e.g.*, immersion in flow-water tanks, application of ozone, UV sterilization; [14]). Hence, robust technologies and procedures are required for a faster and more reliable monitoring of cockles’ bacteriome. This can foster the understanding of environmental-driven shifts in bacterial communities colonising cockles, as well as prevent public health problems through early-informed surveillance of microbial pathogenic loads.

A feasible approach to analyse host-associated bacterial communities relies on DNA-based advanced techniques, which provide a comprehensive characterisation of the composition and structure of the community within a short period of time [[15], [16], [17]]. Two molecular techniques often used to that end are denaturing gradient gel electrophoresis (DGGE) and 16S rRNA gene sequencing (*i.e.*, Next Generation Sequencing techniques; NGS) [[18], [19], [20]]. Given the cost- and time-efficiency of the DGGE technique, it is frequently used to get a preliminary overview of the bacterial community structure and dynamics [21], although only the predominant bacteria can be analysed. Once the 16S rRNA gene is mostly present in bacteria, its sequencing has been regularly used to explore the diversity and relative abundance of bacteria in bivalves (some examples of related works are: [8,22,23]). Therefore, the 16S rRNA gene can be potentially exploited as a surrogate to determine the richness of pathogens that may pose a threat to bivalves, human, and overall ecosystem health. The use of 16S rRNA amplicon sequencing, however, may introduce some uncertainties that should be cautiously addressed. The 16S rRNA gene has multiple copies, which may lead to a certain overestimation, as stated by Bourdonnais et al. [24]. Moreover, it is broadly recognised that the choice of the 16S rRNA hypervariable region, as well as of the primer sets used, can significantly influence the microbial communities identified, ending up in distinct microbiome structures and compositions (*e.g.*, Refs. [25,26]). Nevertheless, the 16S rRNA amplicon sequencing is still considered a valuable approach for the fast and in-depth surveillance of the pathogenic bacterial load [17,27] in bivalves, especially if conducted in the Illumina platform, which provides larger libraries [28].

However, both DGGE and NGS molecular techniques only generate accurate results if a high quality and amount of DNA are available [29]. Hence, DNA extraction and purification are decisive steps to retrieve non-contaminated and non-damaged genomic DNA with high molecular weight, enhanced purity and quantity. Additionally, the extraction process should be fast, economical, require minimal effort and logistical planning. In the particular case of cockles, there are specific challenges to extracting bacterial DNA. One of them is the lower amount of available tissues in cockles to proceed with the extraction, and the other regards the abundantly synthesised mucopolysaccharides and additional polyphenolic proteins, which are extracted along with the DNA and can inhibit enzymatic reactions (*e.g.*, polymerase chain reaction (PCR)) [30]. As such, a fine-tuned selection of the most efficient method to extract bacterial DNA from cockles is necessary.

Currently, there are many commercial DNA extraction kits available to obtain bacterial DNA from different organisms or sample types. Although most of them were not originally designed to be used in bivalves, they were previously applied to extract microbial DNA from different tissues and/or parts (*e.g.,* gills, gut, gonad, digestive gland) of the oysters *Crassostrea gigas* [[31], [32], [33]] and *Crassostrea virginica* [34,35]; the mussels *Choromytilus chorus* [36], *Mytilus coruscus* [37], *Mytilus edulis* [35], *Mytilus galloprovincialis* [8,32,38], *Cyclonaias kieneriana*, *Fusconaia cerina*, *Lampsilis ornata* and *Obovaria unicolor* [39] (*cf*. Table 1). The majority of the used kits are intended for extracting microbial DNA from soil samples (*e.g.*, DNeasy PowerSoil, FastDNA Soil, MoBio PowerSoil DNA isolation, MoBio PowerSoil DNA, FastDNA™ Spin Kit for Soil, E.Z.N.A. Soil DNA, MoBio PowerMax soil), given their enhanced robustness to extract inhibitor-free DNA with appropriate amounts for downstream molecular applications. Indeed, soil extraction kits like the DNeasy PowerSoil kit have been broadly recommended for DNA recovery from different types of samples, with the aim to standardise or harmonise the extraction procedures [43,44]. Some studies also used kits suitable for blood (*e.g.*, AllMag™ Blood DNA, High Pure PCR Template), tissue, stool and gut (*e.g.*, QIAamp PowerFecal DNA, ZymoBIOMICS DNA), and biofilms (*e.g.,* ZymoBIOMICS DNA, Power Biofilm DNA) (*cf*. Table 1). Nevertheless, the extraction of cockle-associated bacterial total-community DNA is considerably overlooked. Moreover, the available studies for other bivalves than cockles only rarely use the same kit, which brings major difficulties in fine-tuning the efficiency of DNA extraction or making the comparison of results. In fact, several studies focusing on kit comparisons have underlined the influence of the “kitome” (*i.e.*, contamination from the kit components or extraction process) on the microbial diversity profiles (*e.g.,* Refs. [[45], [46], [47]]). Thereby, the DNA extraction kits may not perform equally on different systems, hence reinforcing the importance of selecting the most appropriate one to extract bacterial DNA from the gut of cockles.

**Table 1:** List of DNA extraction kits that have been used to retrieve microbial DNA from different bivalve species and sample types, being pointed out the downstream applications for which the kits were originally designed. HTS – High-throughput sequencing. NGS – Next Generation Sequencing.

Bivalve species	Samples used	DNA Extraction Kit	Brand	Recommended sample	Downstream applications	Reference
**Clams**						
*Paphies australis*	Siphons & digestive glands	DNeasy PowerSoil Pro	Qiagen	Soil	PCR, HTS	[40]
**Mussels**						
*Choromytilus chorus*	Gut	DNeasy PowerSoil	Qiagen	Soil	PCR, HTS	[36]
*Cyclonaias asperata*	Gut	MoBio PowerSoil DNA	MoBio Laboratories	Soil	PCR, qPCR, NGS	[39]
*Fusconaia cerina*	Gut	MoBio PowerSoil DNA	MoBio Laboratories	Soil	PCR, qPCR, NGS	
*Lampsilis ornata*	Gut	MoBio PowerSoil DNA	MoBio Laboratories	Soil	PCR, qPCR, NGS	
*Obovaria unicolor*	Gut	MoBio PowerSoil DNA	MoBio Laboratories	Soil	PCR, qPCR, NGS	
*Mytilus galloprovincialis*	Gills, hemolymph, digestive glands, foot, stomach	DNeasy PowerSoil	Qiagen	Soil	PCR, HTS	[8]
*Mytilus galloprovincialis*	Digestive gland, gill, foot, mantle	ZymoBIOMICS DNA	Zymo Research	Microbes, feces, soil, biofilms, water	PCR, HTS	[41]
*Mytilus galloprovincialis*	Gut	MoBio PowerSoil DNA	MoBio Laboratories	Soil	PCR, qPCR, NGS	[38]
*Mytilus galloprovincialis*	Haemolymph and digestive gland	High Pure PCR Template	Roche Diagnostics	Blood, cells, bacteria, yeasts	PCR, restriction enzyme reactions	[32]
*Mytilus edulis*	Gut	Power Biofilm DNA	MoBio Laboratories	Biofilm	PCR, qPCR, NGS	[35]
*Mytilus coruscus*	Gut	FastDNA^TM^ Spin Kit for Soil	MP Biomedicals	Soil	PCR, HTS	[37]
**Oysters**						
*Crassostrea gigas*	Digestive glands	E.Z.N.A. Soil DNA	Omega Bio-Tek	Soil	PCR, HTS	[33]
*Crassostrea gigas*	Gut	E.Z.N.A. Soil DNA	Omega Bio-Tek	Soil	PCR, HTS	[23]
*Crassostrea gigas*	Feces	QIAamp PowerFecal DNA	Qiagen	Stool, gut, biosolids	PCR, HTS	[42]
*Crassostrea gigas*	Gills, digestive glands, muscle tissues	AllMag™ Blood DNA	Allrun	Blood	PCR, other enzymatic reactions	[31]
*Crassostrea gigas*	Haemolymph, digestive gland	High Pure PCR Template	Roche Diagnostics	Blood, cells, bacteria, yeasts	PCR, restriction enzyme reactions	[32]
*Crassostrea virginica*	Gut, gill, mantle tissue, haemolymph, pallial fluid, inner shell	Qiagen Allprep PowerFecal DNA/RNA Kit	Qiagen	Stool, gut, biosolids	PCR, HTS	[22]
*Crassostrea virginica*	Stomach and gut	MoBio PowerMax soil	MoBio Laboratories	Soil	PCR, qPCR, NGS	[34]
*Crassostrea virginica*	Gut	Power Biofilm DNA	MoBio Laboratories	Biofilm	PCR, qPCR, NGS	[35]

As such, we hypothesised that the commercial kits may provide differential efficiencies regarding the recovery and suitability of the bacterial DNA from cockles’ guts for downstream molecular analyses. Thereby, one of our goals was to test the efficiency of five commercial kits on the quality and amount of microbial DNA extracted from the gut of the common cockle, the wild *Cerastoderma edule* Linnaeus, 1758. For that purpose, we have selected the kits that were previously used to recover microbial DNA from other bivalves and that are normally recommended for the extraction of microbial DNA from different sample types, *i.e.*, stool, gut, or cells (FastDNA® Spin kit, hereinafter FastDNA; QIAamp PowerFecal DNA, PowerFecal; ZymoBIOMICS™ DNA Miniprep, hereinafter Zymo), and soil samples (E.Z.N.A.® Soil DNA, hereinafter E.Z.N.A.; DNeasy PowerSoil Pro, hereinafter DNeasy). Besides these features (*cf*. Table 1), the selection of the kits also took into consideration their increased robustness to remove inhibitors from the samples as to enhance the reliability of downstream molecular analyses (*i.e.,* PowerFecal, E.Z.N.A., and DNeasy kits), and to extract DNA from Gram-positive bacteria (*i.e.*, PowerFecal and DNeasy kits).

As a second goal, we have analysed the applicability or integrity of the extracted DNA to study variations in the structure of the bacterial community, through two sequentially applied molecular techniques, the DGGE and Illumina MiSeq sequencing of the 16S rRNA amplicons. Our intent is to provide a technical basis for contracted laboratories, technicians, and researchers willing to have a grounded selection of a commercial kit according to their needs, without requiring additional methodological steps such as sample pre-processing, cell lysis, and the removal of inhibitors. These methodological additions usually demand the use of expensive reagents and materials, whose acquisition and/or use may be financially or timely impractical. The final outcomes of our study can undoubtedly provide technological advice for optimal bacterial DNA extraction from cockles' guts. This will support future research studies on cockle-bacteriome interactions, but may also help regulators in microbiological surveillance programmes, as well as in decision-making processes of cockles' harvest interdictions, which usually have a serious impact on cockles’ production value chains and commercialization.

## 2 Materials and methods

### 2.1 Collection of cockles and gut sample preparation

The wild cockle *C. edule* was harvested during the autumn (September) of 2021 in Canal de Mira (40°37′21.5″N, 8°44′18.2″W) of the Lagoon of Aveiro, Aveiro, Portugal. The collected cockles were placed in a sterile plastic bag and transported on ice to the laboratory. Under aseptic conditions, the guts from a batch of 39 cockles were dissected, pooled, and stored at −20 °C until DNA extraction. The batch of pooled and homogenised guts was weighed to prepare a total of 24 identical replicates (5 replicates *per* kit, except for E.Z.N.A. kit in which only 4 replicates were considered due to the limited amount of guts). Thereby, the same batch of replicates was extracted using the 5 kits, in order to reduce the variability between different replicates or organisms.

### 2.2 DNA extraction and analysis

The total genomic DNA (gDNA) was extracted from gut samples (amount between 199.8 ± 30.71 and 236.6 ± 35.76 mg) using the five commercial DNA extraction kits, namely E.Z.N.A. (Cat. No. D5625; Omega Bio-tek, Norcross, USA), FastDNA (Cat. No. 116540600; MP Biomedicals, Southern California, USA), DNeasy (Cat. No. 47016; Qiagen, Hilden, Germany), PowerFecal (Cat. No. 12830-50; Qiagen, Hilden, Germany), and Zymo (Cat. No. D4300T; Zymo Research, Irvine, USA), following manufacturer's instructions (*cf*. Table S1). The main distinctive features between the 5 kits are outlined in Table 2. All extracted DNA samples were eluted in 50–100 μL of elution buffer or DNase/RNase-free water supplied by each kit (Table 3). The integrity of the gDNA and its eventual contamination by RNA [29] were checked through electrophoresis on a 1 % agarose gel using Lambda DNA/*Hin*dIII marker as a DNA ladder. The quality of the extracted DNA was determined in a NanoDrop™ spectrophotometer using ultrapure water as a blank [38]. The quantification of dsDNA was performed with Qubit 4 Fluorometer (Invitrogen) through the use of Qubit dsDNA BR Assay Kit (Invitrogen).

**Table 2:** Distinctive characteristics of the application, procedures, and materials recommended in the five DNA extraction kits herein selected. gDNA – genomic DNA; preps – preparations or samples; NGS – Next Generation Sequencing.

Characteristics	DNA extraction kits				
	E.Z.N.A.	FastDNA	DNeasy	PowerFecal	Zymo
Sample type or matrix	Soil	Plants, animal tissues, yeast, bacteria, algae, fungi, others	Soil	Stool, gut, biosolids	Bacteria, fungi, water, protozoans, algae, virus, faeces, soil, biofilms
Source of the isolated gDNA	Bacteria, fungi, yeast, algae	Plants, animals, bacteria, yeast, algae, fungi	Bacteria, fungi	Bacteria, fungi	Bacteria, fungi, protozoans, algae
gDNA isolation from Gram-positive?	Yes	No	No	No	No
Downstream application	PCR, Southern blot, NGS	PCR and other applications	PCR, NGS, enzymatic digestion assays	PCR, NGS, enzymatic digestion assays	qPCR, 16S rRNA & shotgun sequencing
Cell lysis	Mechanical & Chemical	Mechanical & Chemical	Mechanical & Chemical	Mechanical & Chemical	Mechanical & Chemical
Bead material and size	Glass, 0.1∼0.2 mm	Garnet (flakes), 0.56-0.7 mm	Ceramic, 0.1-0.5 mm	Dry Garnet, 0.7 mm	BashingBeads of chemically-inert material, 0.1-0.5 mm
Cell lysis methods	Vortex 5’; incubation at 70 °C, 10’	Vortex for 40 s	Vortex (Genie® 2) 10’	Heating at 65 °C 10’; vortex (Genie® 2) 10’	Vortex 10’
Recommended mass (mg)	100-200	100-200	≤ 250	≤ 250	≤ 250
Approximate cost	193€ / 50 preps	363€ /100 preps	420€ / 50 preps	417€ / 50 preps	435€ / 50 preps

**Table 3:** Average quality (measured by the *A*_*260/280*_*ratio*; in NanoDrop™), concentration (*[DNA]*; measured in Qubit Fluorometer), and amount of DNA extracted from *Cerastoderma edule* gut mass, are presented along with the volume of elution buffer used *per* extraction kit. Values are presented as mean ± standard deviation. Different letters (in superscript) highlight kits showing significantly different performance in terms of DNA concentration and recovery from gut samples, as *per* the one-way ANOVA outcome (*P* < 0.05; *cf*. Table S2).

DNA extraction kits	Sample mass used (mg)	Elution buffer (μL)	A_260/280_ ratio	[DNA] (ng μL^−1^)	Amount DNA/mg gut (ng mg ^−1^)
E.Z.N.A.	200.0 ± 81.65	50	1.91 ± 0.02	62.1 ± 22.03^a^	19.2 ± 14.72^a^
FastDNA	200.0 ± 00.00	100	1.70 ± 0.12	120.3 ± 37.04^b^	60.2 ± 18.52^b^
DNeasy	228.3 ± 30.08	50	1.90 ± 0.02	66.9 ± 6.86^a^	15.2 ± 2.62^a^
PowerFecal	236.7 ± 35.76	50	1.95 ± 0.20	5.5 ± 3.77^c^	1.08 ± 0.629^a^
Zymo	199.8 ± 30.71	50	2.04 ± 0.26	3.8 ± 1.33^c^	0.94 ± 0.277^a^

### 2.3 Bacterial community analysis by DGGE

#### 2.3.1 Amplification of 16S rRNA gene by PCR

The hypervariable V3-V4 region of the 16S rRNA gene was amplified with the following primers: 338F (5′-G-ACTCCTACGGGAGGCAGCAG-3′) and 518R (5′-ATTACCGCGGCTGCTGG-3′), with a GC clamp attached to the forward primer [18]. The PCR mixture had a total volume of 25 μL with 12.5 μL of DreamTaq DNA Polymerase (Thermo Fisher Scientific, Waltham, Massachusetts, EUA), 0.75 μL of each primer, 10 μL of water, and 1 μL of DNA template. PCRs were conducted in a T100™ thermal cycler (Bio-Rad Laboratories, CA, USA). The amplification of the bacterial DNA started with an initial denaturation step of 95 °C for 5 min, followed by 35 cycles of denaturation at 92 °C for 30 s, annealing at 55 °C for 30 s, and elongation at 72 °C for 5 min. The final extension lasted 10 min at 72 °C. Aliquots of 2 μL of the PCR products were evaluated on a 1 % agarose gel electrophoresis (Fig. S1).

#### 2.3.2 DGGE run

DGGE analysis was performed with the DcodeTM Universal Mutation Detection System (Bio-Rad Laboratories, Hercules, CA, USA) as described in Almeida et al. [48]. The PCR products obtained previously were loaded on a 8 % vertical polyacrylamide gel with a linear denaturing gradient ranging from 35 % to 60 % (100 % denaturant contains 7 M urea and 40 % formamide). The electrophoresis run was performed at 60 °C, initially at 20 V for 15 min, and then at 75V for 16 h, in 1x TAE buffer. As a reference marker, a sample composed of 12 bands, and stored at the laboratory was used. After electrophoresis, the DGGE gel was stained as described in Henriques et al. [21]. The gel image was obtained using the Molecular Imager® Gel DocTM XR^+^ System (Bio-Rad Laboratories, CA, USA). The DGGE band matrix resulted from the analysis of the DGGE profiles using BioNumerics Software (Applied Maths, Ghent, Belgium), following the manufacturer recommendations.

### 2.4 Bacteriome analysis by illumina high-throughput sequencing

The five DNA samples obtained from each DNA extraction kit were pooled and prepared for Illumina MiSeq high-throughput sequencing (performed by Eurofins Genomics) targeting V3-V4 hypervariable regions of the 16S rRNA gene (forward primer: 5′-TACGGGAGGCAGCAG-3’; reverse primer: 5′-CCAGGGTATCTAATCC-3′). The obtained sequences were processed according to Eurofins protocols and platforms. Briefly, reads were demultiplexed according to their index sequences, and quality-filtered to remove the primer target regions or to trim out low-quality ends. Reads with overlapping forward and reverse ends were merged, filtered according to the expected length and the variations of the target region. Reads containing ambiguous bases were removed. The detection and removal of chimeras was performed through the *de-novo* algorithm of UCHIME [49]. At this point, the set of high-quality reads obtained (*i.e.*, chimera-removed sequences) was used as input data for the derivation of Operational Taxonomic Units (OTUs) using Minimum Entropy Decomposition (MED), partitioning the marker gene datasets into OTUs [50]. The MED procedure identified and filtered random "noise" in the dataset, considering a minimum substantive abundance threshold of 10, and a maximum variation allowed of 20. The taxonomy assignment to each OTU was achieved by DC-MEGABLAST alignments of cluster representative sequences to the NCBI database (NCBI_nt (/dbdir/nt._ltered.fa); released on 2020-03-02). The most specific taxonomic identity of each OTU was inferred from the set of best-matching reference sequences, considering the lowest common taxonomic unit of all best hits. A sequence identity threshold of 70 % across at least 80 % of the representative sequence was a minimal requirement for considering reference sequences. All taxonomic units with less than 0.1 % of reads were collapsed in the category "Other". If the representative sequence of an OTU had no significant database match, no taxonomic unit could be assigned. All reads of these unclassified OTUs were allocated to the category “Unclassified”. OTUs that did not match the expected clade were filtered out. Additional processing of OTUs and the respective taxonomic assignments was done in QIIME software package (version 1.9.1). The lineage-specific 16S rRNA gene copy number was used to normalise bacterial taxonomy unit abundances [51].

### 2.5 Statistical analysis

The extraction efficiency was determined by computing the concentration of DNA extracted *per* mass (g) of the sample used [52]. A one-way analysis of variance (one-way ANOVA) followed by the *post-hoc* Tukey test (α = 0.05) was applied to the concentration of DNA, amount of DNA recovered *per* mg of gut, and DNA extraction efficiency for the five extraction kits, in order to discern potential significant differences between the kits.

The matrix of DGGE bands was analysed using the PRIMER version 6.0 software (Primer-E Ltd., Plymouth, UK). A multivariate analysis of DGGE band matrix using the Bray-Curtis similarity distance matrix was performed, namely, (i) cluster analysis, (ii) principal coordinate analysis (PcoA), (iii) analysis of similarities (ANOSIM), and (iv) permutational multivariate analysis of variance (PERMANOVA), being ANOSIM and PERMANOVA based on 999 permutations [53]. A one-way ANOVA was used to compare the DGGE profiles obtained for PCR amplicons from each kit. A significance level of 0.05 was considered.

Regarding the analysis of the high-throughput sequencing data, the structure of the bacterial communities was examined by PCoA based on the Bray-Curtis coefficient of similarity of the OTUs. The OTUs abundance and diversity *per* sample were assessed through the calculation of the Pielou's evenness (*J′*), the species richness (*S*), and the Shannon-Wiener diversity index (*H′*) by using the Primer 6.0 software. The heatmap was constructed to show the 30 most abundant OTUs in all samples.

## 3 Results and discussion

The cockle-bacteriome relationship has been quite overlooked, despite its relevance to get a deeper understanding of ecosystem dynamics and food web interaction in estuarine environments, and a safer exploitation of ecosystem services associated with this bivalve. In any case, a key condition to digging into the gut bacteriome of cockles through NGS or other molecular tools is the efficient recovery of microbial DNA. A sufficient amount of high-quality DNA is required to obtain a representative composition of the microbial community. Thereby, the present study compared the efficiency of five commercial kits to extract bacterial DNA from *C. edule* gut for downstream molecular and genomic analyses.

### 3.1 Analysis of the DNA purity and concentration

Depending on the kit used, different levels of DNA quality and yield were obtained (*cf*. Table 3and S2). This outcome is in line with previous studies focusing on the influence of the extraction method on the DNA purity and quantity retrieved from different biological niches and sample types [54]. The level of DNA purity has been commonly characterised by a A_260/280_ ratio of ∼1.8 [55,56]. The results presented in Table 3 show that for most of the kits tested, the mean value of the A_260/280_ ratio was close to 1.8, hence suggesting that the extracted gDNA from *C. edule* gut samples was generally of good quality. The Zymo kit, however, provided the highest average value of the A_260/280_ ratio (2.04 ± 0.26). This could be indicative of RNA contamination in the sample, whose absorbance cannot be distinguished from that of DNA in the spectrophotometer reading [57]. On the other hand, the FastDNA kit retrieved the lowest average value of A_260/280_ ratio (1.70 ± 0.12), being also observed a smear effect in the electrophoretic run, thereby indicating a certain degradation of the gDNA. Normally, values below 1.8 are also attributed to traces of RNA, proteins, phenol, and other contaminants [29,58], which may constrain the accuracy of molecular analyses. As such, based on a purity-wise analysis, the DNA extraction kits that provided the best result were the E.Z.N.A and DNeasy kits.

In a general view, except for PowerFecal and Zymo kits, the obtained DNA concentrations were sufficient, given the usual minimum requirements (5–10 ng μL^−1^) for Illumina sequencing [59]. The average DNA concentration resulting from the Zymo kit was significantly lower compared to all kits, except the PowerFecal kit (Table 3 and S2). According to Xue et al. [59], DNA concentrations close to those achieved with the Zymo kit did not influence the microbiome profiling of shrimp larval samples. By contrast, our results did not corroborate this conclusion, since the Zymo kit provided the lowest values of richness and diversity indices for cockles’ gut samples (*cf*. section 3.3).

In addition, it is known that a contaminated DNA sample, as is seemingly the case with the FastDNA sample, may lead to inaccurate DNA concentration measurements [60,61]. As such, with regard to the DNA concentration, the best results for the gut samples of cockles were once again obtained with the DNeasy kit, since the E.Z.N.A. kit provided a lower average of DNA concentration with increased variability between replicates (higher standard deviation).

**Fig. 1:**
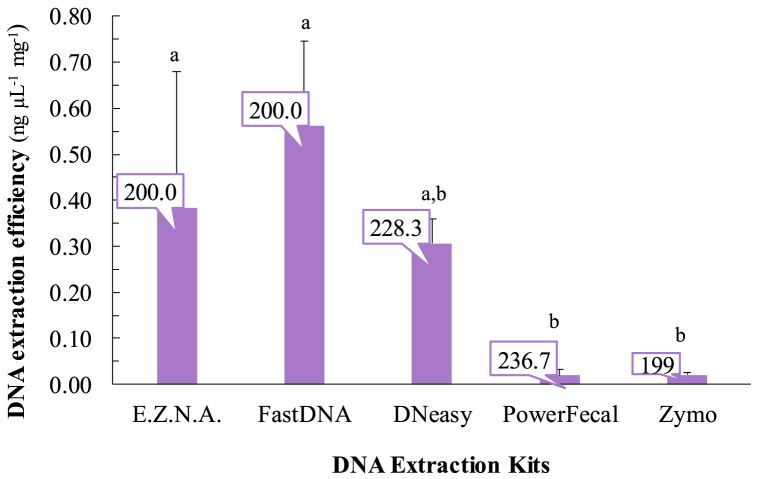
DNA extraction efficiency according to the mass (mg; labels on the top of the respective bars) of *Cerastoderma edule* gut sample used in the different DNA extraction kits. Error bars represent the standard deviation. Different letters highlight kits showing significantly different DNA extraction efficiencies, following the one-way ANOVA outcome (*P* < 0.05; *cf*. Table S2).

Despite the variable amounts of DNA recovered *per* mg of gut by the five kits, only FastDNA showed a statistically different performance in regard to the other four kits (Table 3 and S2). A similar pattern was observed for the DNA extraction efficiencies represented in Fig. 1, according to which the kits can be ordered by their increasing extraction efficiency as follows: PowerFecal ≈ Zymo < DNeasy < E.Z.N.A. < FastDNA.

It is largely accepted that an inefficient extraction of microbial DNA can blur the representativeness of the bacteriome associated with cockles. This may not only constrain the understanding of cockle-bacteriome relationships within an ecological context, but may also lead to over- or underestimation of the presence of pathogenic species/strains of bacteria, which are a concern for public health protection and food safety. Therefore, it is highly relevant to assess the potential influence of different factors on the performance of DNA extraction kits. For instance, the sample type may interfere with the extraction efficiency of some kits. The FastDNA, PowerFecal and Zymo were initially designed for tissues and/or stool, whereas E.Z.N.A. and DNeasy kits were optimised for soil samples (Table 2). In fact, FastDNA provided the highest DNA concentrations (though with a certain level of contamination), but PowerFecal and Zymo retrieved the lowest DNA yields and extraction efficiency (Table 1, Table 3 and S2).

Another interfering factor, besides the sample type, is the cell lysis method. The DNA concentration may be strongly affected by the efficiency of the cell disruption procedure for releasing the DNA [62]. The compared kits varied slightly in the techniques used to lyse cells, although they all apply mechanical and chemical methods (Table 2 and S1). Some studies have reported that the mechanical bead-beating step, which is recommended and was used in the five kits, can increase the DNA concentration extracted from faecal [63,64] and stomach samples [65]. However, Bürgmann et al. [66] showed that the amount and type of beads affected the DNA yield as well.

Another procedural issue is the incubation temperature, which appears to affect proteinase K activities. In a study conducted by Qamar et al. [67], at 50 °C the enzyme digested the contaminating proteins, but at 55 °C a negative effect was observed. Nevertheless, 75 °C led to low quantities of DNA [66]. Bitskinashvili et al. [68] also indicated that the heat treatment affected the DNA integrity, which in turn may affect the DNA quality at the end.

Despite all the factors that may potentially interfere with DNA extraction, their actual influence can only be confirmed upon the realization of future studies designed for assessing their individual effect under the same conditions. Yet, in the present study, it is possible to verify that, based on the quality and quantity of DNA retrieved from cockles’ gut samples, the DNeasy kit was the one providing better results.

### 3.2 Bacterial community structure analysed by DGGE

The generated DGGE profile is presented in Fig. S2. In general, a band pattern could be identified for most kits tested. The low intensity and low number or absence of DGGE bands observed in replicates extracted with the FastDNA kit could have resulted from the lower quality of the input DNA (*cf*. Table 3) [69] or the presence of DNases in the sample, due to a possible limited capacity of the kit to inhibit their activity. The presence of PCR inhibitors in the sample can in turn prevent the correct amplification of the DNA fragments, thereby introducing biases in the molecular profiling and sequencing results [70,71]. Moreover, the samples from the other four kits (*i.e.*, E.Z.N.A., DNeasy, PowerFecal and Zymo) exhibited a lot of high intensity bands, which was notably evident for the E.Z.N.A. and DNeasy kits (Fig. S2).

**Fig. 2:**
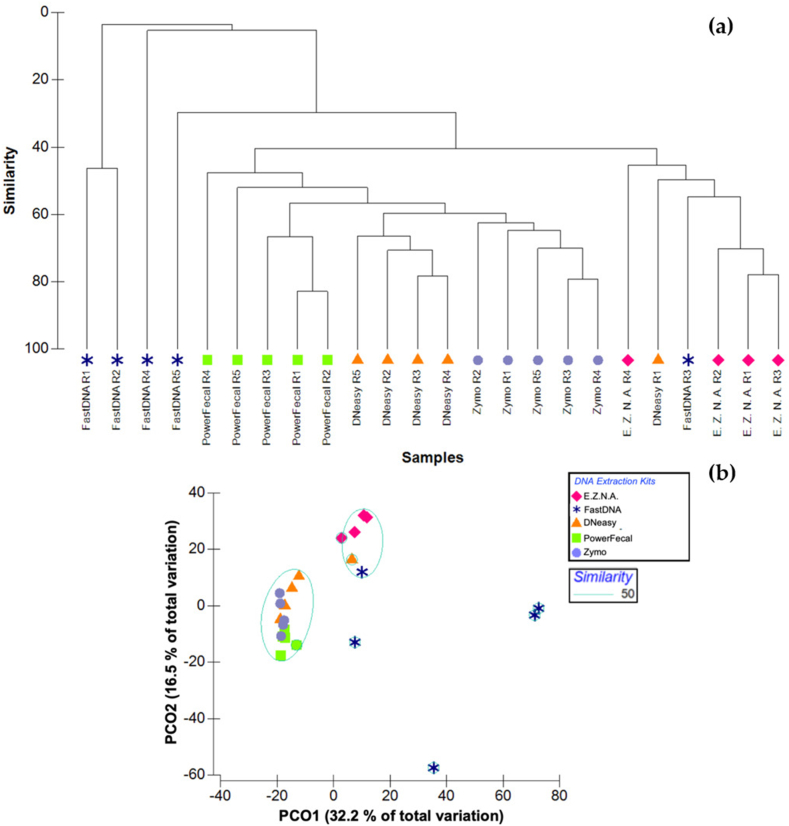
**(a)** Dendrogram of Bray-Curtis distance matrix obtained from the banding pattern in DGGE profiles using cluster analysis; **(b)** Principal Component Analysis (PCoA) of the PCR-DGGE of the *Cerastoderma edule* gut using Bray-Curtis dissimilarity matrices, representing the similarity between all samples analysed.

With the aim of analysing the similarity of the band patterns among kits, a dendrogram was constructed using cluster analysis. Fig. 2a shows the similarity between three main groups of samples: (i) the most dissimilar group is constituted by four replicates of the FastDNA kit; (ii) a second major group consists of DNA samples obtained with the PowerFecal, DNeasy and Zymo kits; and (iii) the last one mostly joins the E.Z.N.A.-extracted DNA replicates. This trend is also evident in the PCoA, according to which two main groups with 50 % similarity were separated (Fig. 2b). Broadly, it was observed that there was a high similarity in the band profiles between replicates of each kit. In fact, the DNA samples extracted with DNeasy and Zymo kits presented the highest intra-similarity (*ca.* 60 % similarity between replicates) (Fig. 2a). This result is in line with the purity level of the DNA template obtained with the two latter kits (Table 3).

The significance of the similarities observed in the DGGE band patterns between the five DNA extraction kits was evaluated by an ANOSIM and PERMANOVA, which outcomes are presented in Table 4. According to Ramette [53], the *R* value is used to evaluate the degree of separation within and between treatments (*i.e., R* = 0, high similarity with no separation of communities' structure; *R* < 0.25, barely separable; 0.25 < *R* < 0.5, moderately separable; *R* > 0.5, separable; *R* = 1, no similarity on the separation of communities’ structure). Although the global *R* showed that there are significant differences (*P* value always <0.05) between the patterns of bands obtained with the different kits (*Global-R* = 0.512), the pairwise analysis revealed different results depending on the kits. A previous study had also found significant differences between the profiles of the bacterial community associated with *Mytilus edulis* mussels, depending on the DNA extraction method used (global *R* = 0.641; [72]). The DGGE profiles of E.Z.N.A. replicates were significantly separated from those of the DNeasy, PowerFecal and Zymo kits (*R* > 0.5; *P*: 0.005–0.015), similarly to the separation computed for DNeasy *vs.* PowerFecal samples (*R* = 0.656; *P* = 0.013). In turn, the FastDNA samples were barely (from E.Z.N.A kit samples; *R* = 0.175; *P* = 0.018) to moderately (DNeasy, PowerFecal and Zymo kits; *R*: 0.440–0.500; *P*: 0.006–0.012) separated from all other kits. Overall, the structure of the communities in E.Z.N.A. samples was more distinctive comparatively to that obtained with the other kits (higher *R* values).

**Table 4:** Summary of the one-way ANOSIM statistical analysis (*R*) based on the Bray-Curtis matrix computed for DGGE band profiles of the DNA extracted with five different kits (E.Z.N.A., FastDNA, DNeasy, PowerFecal and Zymo). Significant differences (*P value* ≤ 0.05) are presented in bold, and they were computed upon the application of a PERMANOVA analysis.

Groups	*R*	*P* value
E.Z.N.A., FastDNA	0.175	**0.018**
E.Z.N.A., DNeasy	0.806	**0.015**
E.Z.N.A., PowerFecal	0.994	**0.010**
E.Z.N.A., Zymo	0.938	**0.005**
FastDNA, DNeasy	0.440	**0.006**
FastDNA, PowerFecal	0.496	**0.012**
FastDNA, Zymo	0.500	**0.007**
DNeasy, PowerFecal	0.656	**0.013**
DNeasy, Zymo	0.448	**0.015**
PowerFecal, Zymo	0.420	**0.009**
**Global-*R***	0.512	-

The 5 replicates of each individual DNA extraction kit were pooled together, since their bacterial community structure was highly similar (*i.e.*, no observed significant differences between replicates, *P* > 0.05; *cf*. Table S3). The pooled replicates of each individual kit were then analysed by high-throughput sequencing. Notwithstanding, the FastDNA kit replicates were not sequenced due to the reduced quality and integrity of the DNA, and the limited performance of the kit to recover a representative structure of the bacterial community associated with *C. edule* gut. Therefore, these DNA samples were withdrawn from the remaining analyses.

### 3.3 Analysis of the cockle gut bacteriome by 16S rRNA gene sequencing

The sequencing of 16S rRNA amplicons from the four gut-bacteriome DNA samples (one pooled sample *per* extraction kit) yielded 277,363 read sequences, which resulted in a total of 337 OTUs assigned to *taxa* (*cf*. Table S4 for the relative abundance of bacterial OTUs identified in the *C. edule* gut *per* kit).

The highest OTU number (*S*) was obtained with the E.Z.N.A. kit (109 OTUs; *ca.* 32 % of total OTUs), followed by the DNeasy (63 OTUs; 19 % of all OTUs), PowerFecal (67 OTUs; 20 % of all OTUs), and Zymo (17 OTUs; 5 % of total OTUs) kits (Table 5). Based on the *H′* Shannon-Wiener diversity index, both PowerFecal and DNeasy kits enabled higher diversity, compared with the E.Z.N.A. and Zymo kits. As for the uniformity of samples (according to Pielou's evenness index, *J′*), the kits can be arranged as follows: PowerFecal > DNeasy = Zymo > E.Z.N.A. Overall, based on the analysis of alpha diversity, the DNeasy and PowerFecal kits presented higher values for the three metrics.

**Table 5:** Diversity indices: Species richness (number of OTUs, *S*), Pielou's evenness index (evenness, *J′*), and Shannon-Wiener diversity (diversity, *H′*), calculated for each kit used to extract bacterial DNA from the *Cerastoderma edule* gut.

DNA Extraction Kits	*S*	*J’*	*H’* (ln)
E.Z.N.A.	142	0.68	3.36
DNeasy	129	0.74	3.61
PowerFecal	120	0.79	3.80
Zymo	91	0.74	3.32

**Fig. 3:**
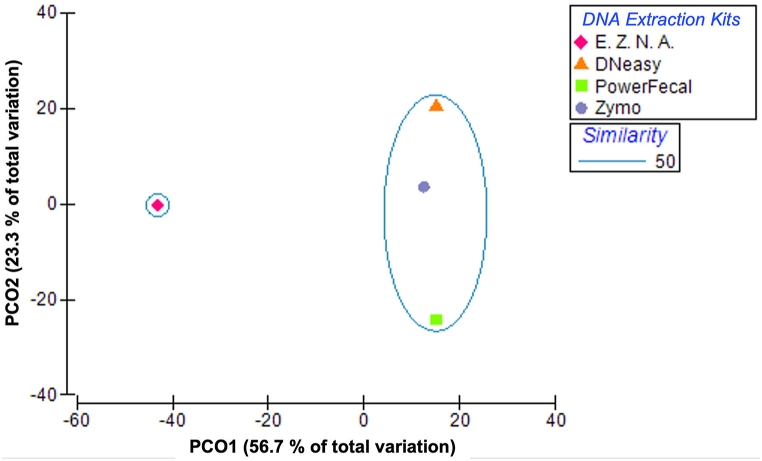
PCoA using Bray-Curtis similarity matrices representing the correlation between the OTUs detected in *Cerastoderma edule* gut upon the use of four different DNA extraction kits.

The PCoA analysis of the OTUs separated E.Z.N.A. from the other DNA extraction kits, hence evidencing a bacterial community with distinctive structure and abundance (Fig. 3). In turn, the samples from DNeasy, PowerFecal, and Zymo presented 50 % similarity, sharing 16 OTUs (*i.e.*, 5 % of the total number of OTUs derived for all kits; Table S4).

**Fig. 4:**
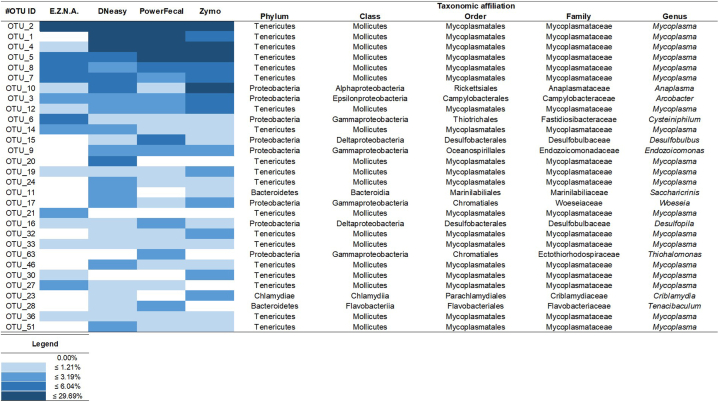
Heatmap showing the 30 predominant OTUs in regard to four DNA extraction kits, with their respective taxonomic affiliations. The colour code represents the relative abundance (%) of OTUs. The OTU list is provided in the supplementary material as Table S4, to enable further representation of the genera diversity obtained with each extraction kit.

A heatmap of the 30 most abundant OTUs was constructed for comparative analysis (Fig. 4; Fig. S3 shows the heatmap of the 30 top-represented OTUs without considering *Mycoplasma* spp. OTUs). The pattern of the core bacteriome differed between the extraction kits when considering the number of OTUs and their respective relative abundances. Based on Fig. 4, the E.Z.N.A. sample presented 18 OTUs of the 30 dominant ones, the DNeasy presented 27 OTUs, and the PowerFecal and Zymo kits had 25 OTUs each. In global, the phyla represented in the gut bacteriome of cockles were mainly Tenericutes (class Mollicutes), Proteobacteria (classes Alphaproteobacteria, Gammaproteobacteria, Deltaproteobacteria, Epsilonproteobacteria), Bacteroidetes (class Flavobacteriia), and Actinobacteria (Fig. 4 and S3), which is in agreement with previous studies unravelling the microbiome associated with oysters (*e.g.*, Ref. [22]), mussels (*e.g.,* Refs. [8,73,74]), and clams (*e.g.*, Ref. [75]). At the genus level, the predominant OTUs were affiliated with *Mycoplasma*, followed by *Anaplasma*, *Acrobacter*, and *Cysteiniphilum* genera. Despite this, the most abundant OTUs in the entire community were assigned to *Mycoplasma*, which represented *ca.* 50 % of the total relative abundance (Table S5).

The capacity of a kit to recover Gram-positive bacteria is a key selection criterion [62], given the difficulty of disrupting their thick and rigid cell walls [76]. In total, 18 OTUs (*ca.* 5 % of the total number of OTUs; Table S6) were affiliated with Gram-positive bacteria, corresponding to 2.23 % of the total relative abundance of the entire bacterial diversity, whereas the remaining 319 OTUs (95 %) were assigned to Gram-negative bacteria. The community of Gram-positive bacteria was mostly assigned to the phyla Actinobacteria, Firmicutes, and Tenericutes (Table S6). The best-performing kit for the recovery of DNA from Gram-positive bacteria was the PowerFecal kit (11 OTUs assigned, 3 % of total OTUs), followed by DNeasy (8 OTUs, 2 % of total OTUs), Zymo (8 OTUs, representing 2 % of total OTUs), and E.Z.N.A. kits (2 OTUs, representing 1 % of total OTUs) (*cf*. Table S6). Notwithstanding, the Gram-positive OTUs extracted by the DNeasy and Zymo kits showed higher diversity at the genus level compared to the PowerFecal and E.Z.N.A. kits.

Although the E.Z.N.A. kit has an additional step for improving the DNA extraction of Gram-positive bacteria, a reduced number of OTUs was assigned to this gram stain (Table S6). This suggests that the initial steps of mechanical (*e.g.*, bead-beating process) and chemical (addition of cell wall-degrading agents) cell disruption should be longer, and/or consider the addition of (higher concentrations) of phospholipid membrane-lysing enzymes (*e.g.,* lysozyme). Such steps may enhance the efficiency of DNA extraction from Gram-positive bacteria, and reduce the introduction of biases in the analysis of bacteriome [59]. Indeed, several studies reported that different commercial extraction kits may have different efficacies in extracting DNA from Gram-positive *vs.* Gram-negative bacteria, reflecting this result in the amplicon-sequencing output of the 16S rRNA or other marker genes [77,78].

**Fig. 5:**
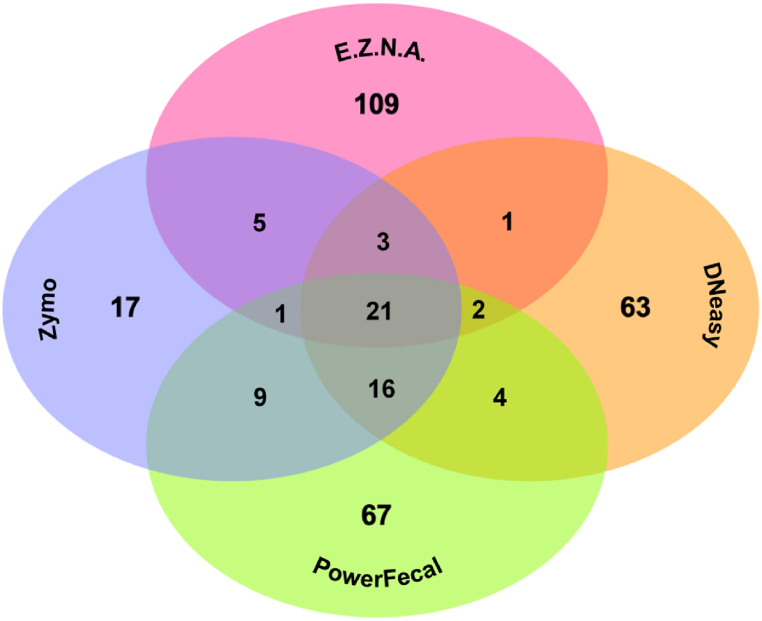
Venn diagram illustrating the number of shared and unique OTUs for the different DNA extraction kits.

Broadly, the sequencing outcomes demonstrated that some of the used kits recovered a similar bacterial composition profile in *C. edule* gut (*e.g.*, PowerFecal and DNeasy kits), while others performed quite differently (*e.g.*, E.Z.N.A.). The samples from all four kits shared 21 OTUs (Fig. 4, Fig. 5), representing 6 % of the total number of reported OTUs and 65.77 % of the total relative abundance of the whole community (Table S5). PowerFecal- and Zymo-extracted DNA shared 47 OTUs (14 % of total OTUs); while the DNeasy kit shared 43 OTUs (13 % of total OTUs) with PowerFecal and 40 OTUs (12 % of total OTUs) with Zymo. Both Zymo (55 OTUs) and PowerFecal (53 OTUs) shared a higher number of OTUs with other kits, followed by DNeasy with a total of 47 shared OTUs (Fig. 5).

**Fig. 6:**
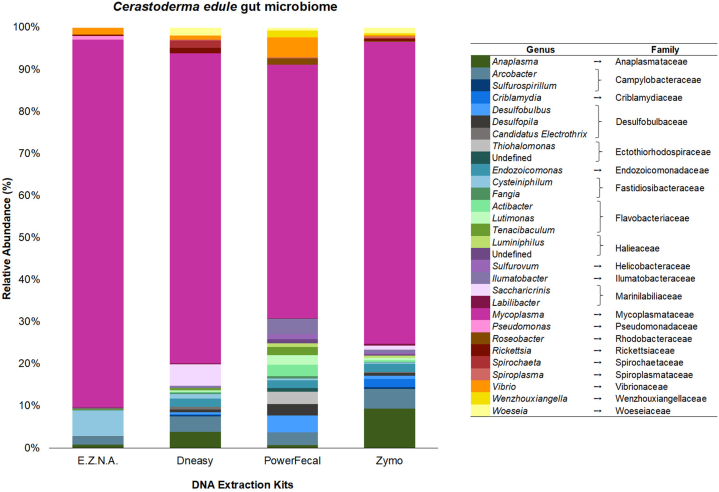
Relative abundance (%) of the top 30 most abundant genera (and respective families) in the *Cerastoderma edule* gut bacteriome (total of 82 genera), following the use of different DNA extraction kits. Genera classified as “undefined” were assigned to the family name.

Overall, a total of 82 genera were derived (Table S7), being the 30 most abundant presented in Fig. 6 (Fig. S4 shows the 30 most abundant genera after removing *Mycoplasma* spp. to get a broader overview of other represented genera). With the E.Z.N.A. kit a predominance of the *Mycoplasma* genus was obtained, which represented 85 % of relative abundance in this sample, followed by *Cysteiniphilum* (6 %), *Arcobacter* (2 %), and *Vibrio* (2 %). With the DNeasy kit were mainly detected *Mycoplasma*, *Saccharicrinis*, *Anaplasma*, and *Arcobacter*, representing respectively 71 %, 5 %, 4 %, and 3 % of relative abundance (Fig. 6). With the PowerFecal kit, it was observed that the bacterial community was mostly composed of *Mycoplasma*, *Vibrio*, *Desulfobulbus*, and *Ilumatobacter* genera, which accounted for 54 %, 4 %, ∼4 %, and 3 % of relative abundance of the sample, respectively. The bacteriome of the Zymo kit was characterised by a higher relative abundance of *Mycoplasma* (70 %), *Anaplasma* (9 %), *Arcobacter* (5 %), and *Criblamydia* (2 %). As abovementioned, the *Mycoplasma* was the most abundant genus, particularly in E.Z.N.A. and DNeasy samples. In addition, the DNeasy also retrieved the dominant genera obtained with the other kits. Globally, the PowerFecal (49) and DNeasy (41) kits allowed the identification of a higher number of genera compared to Zymo (36) and E.Z.N.A. (24). In summary, 8 bacteria genera were isolated by all four kits tested, including *Mycoplasma*, *Anaplasma*, *Arcobacter*, *Cysteiniphilum*, *Vibrio*, and *Desulfopila* (Fig. 6). *Pseudomonas* was uniquely isolated by the E.Z.N.A. kit, whereas the *Thiohalomonas* genus was only retrieved by the PowerFecal kit.

The predominance of *Mycoplasma* has been often linked to the core microbiome of bivalves (*e.g.*, Refs. [22,79,80]), namely in the digestive gland of the mussel *Villosa nebulosa* [73], in the stomach of *Mytilus galloprovincialis* [8], and in the stomach and gut of the oyster *Crassostrea gigas* [34,79]. However, *Anaplasma* was also previously detected in the whole content of *M. galloprovincialis*, including the digestive gland, gills, foot, mantle, and liquid [74]. *Arcobacter* was previously found in the bacteriome of the oyster *Tiostrea chilensis* [81], as well as in the haemolymph of *C. gigas* [15]. In contrast, the bacterium *Cysteiniphilum* was reported in other phylum than Mollusca (*e.g.*, the cnidarian *Acropora cervicornis* [82]), but some species have been recently considered emerging pathogens capable of causing human infections [83]. Therefore, this association underpins the need for more robust and accurate techniques in support of those regularly applied in the surveillance of pathogenic bacteria (*i.e*., enumeration of *E. coli*) in cockles as to secure public health.

On the other hand, the occurrence of sulphur-oxidising (*e.g., Thiohalomonas*) and sulphate-reducing (*e.g.*, *Desulfopila* and *Desulfobulbus*) bacteria has been associated with hypersaline environments with depleted oxygen and high organic loads [[84], [85], [86]], which actually characterise the estuarine sediments from where the cockles were sampled. *Vibrio* is a known pathogen capable of infecting bivalves and impairing the diversity of their core microbiome [87]. However, according to Rasmussen et al. [88], the abundance of *Vibrio* may reduce when *Mycoplasma* sp. is prevalent, which may explain the low extraction of *Vibrio* DNA by some kits.

## 4 Conclusions

In light of the results achieved for the tested kits, we recommend the use of the DNeasy kit for studying *C. edule* gut bacteriome. Although this kit was originally designed for soil samples, it performed well and enabled the recovery of total-community DNA with superior quality, reasonable yield, and extraction efficacy. These are prime and necessary criteria for having enough and suitable DNA template for downstream applications. The DNeasy kit also retrieved a higher abundance and diversity of bacterial communities from the cockles’ gut, as deduced from the analysis of the DGGE profiles and the 16S rRNA gene amplicon sequencing data. From the latter analysis it was also evident that the DNeasy kit recovered a core bacteriome characterised by a high richness, evenness, and diversity of OTUs, besides showing the same mostly-abundant genera also detected with the other extraction kits. Although the PowerFecal kit allowed the identification of a higher number of Gram-positive bacteria, it recovered particularly low DNA yields with reduced extraction efficiency, which may seriously constrain the use of DNA in downstream molecular techniques. The FastDNA™ Spin kit apparently provided the worst outcomes for *C. edule* gut bacteriome studies compared to the other kits tested. The selection of methods to recover total-community bacterial DNA from the gut of bivalves is a very important step. This should be particularly cautious to avoid biases in the bacteriome analysis, which can prevent an accurate assessment of cockle-bacteriome interactions or the surveillance of pathogenic bacteria to avoid foodborne diseases and public health disasters.

Supplementary Materials. Table S1: Major extraction steps instructed by the manufacturer for each of the five DNA extraction kits.; Table S2: Summary of the one-way analysis of variance (*F*) for different parameters associated with the DNA extraction undertaken with the five kits. [DNA]- concentration of DNA; df – degrees of freedom; P value – value of probability.; Table S3: Summary of the one-way ANOVA applied to the DGGE band patterns obtained from the PCR amplicons for the five extraction kits.; Table S4: Relative abundance of bacterial OTUs identified in the *Cerastoderma edule* gut by four DNA extraction kits (E.Z.N.A., DNeasy, PowerFecal, and Zymo). nd – not determined.; Table S5: Relative abundance of 21 bacterial operational taxonomic units (OTUs) identified in the *Cerastoderma edule* gut that are shared by the four DNA extraction kits (E.Z.N.A., DNeasy, PowerFecal, and Zymo).; Table S6: List of operational taxonomic units (OTUs) affiliated to Gram-positive bacteria (being presented the respective Phylum, Class, Order, Family, Genus and Species) extracted from the *Cerastoderma edule* gut by four DNA extraction kits (E.Z.N.A., DNeasy, PowerFecal, and Zymo). nd – not determined.; Table S7: Relative abundance of the bacterial genera associated with *Cerastoderma edule* gut, as recovered by four DNA extraction kits (E.Z.N.A., DNeasy, PowerFecal, and Zymo). nd - not determined. Fig. S1: Example of PCR amplifications run in 1 % agarose gel electrophoresis. The marker (1 kb DNA ladder, ThermoScientific) is in the first lane; the next 5 lanes correspond to the amplicons of the five replicates obtained for one DNA extraction kit.; Fig. S2: DGGE profiles of the bacterial community associated with the *Cerastoderma edule* gut, using five different DNA extraction kits (indicated above the gel picture; 5 replicates were tested *per* extraction kit). MM: molecular marker.; Fig. S3: Heatmap showing the 30 predominant OTUs without *Mycoplasma* spp. in regard to the DNA extraction kit used (E.Z.N.A., DNeasy, PowerFecal, and Zymo). The colour code represents the relative abundance of OTUs (%). Values below 0.1 % of relative abundance are represented in white.; Fig. S4: Relative abundance of the 30 predominant genera without *Mycoplasma* spp. in *Cerastoderma edule* gut, following the application of four DNA extraction kits (E.Z.N.A., DNeasy, PowerFecal, and Zymo).

## Funding

This research was funded by Mar 2020 through PT 2020 and the European Maritime and Fisheries Fund (EMFF) to the SEEBug project (MAR2020-P01M03-0511P). Catarina Lourenço and Ana R. Almeida worked under a fellowship granted by the SEEBug Project. Catarina R. Marques is funded by national funds (OE), through FCT (Foundation for Science and Technology, I.P.) under the project with reference 2022.05268.CEECIND. We are grateful to the financial support to 10.13039/100013239CESAM by FCT/MCTES (UIDP/50017/2020 + UIDB/50017/2020 + LA/P/0094/2020), through national funds.

## Data availability statement

Data is contained within the article or in supplementary material, being sequencing data available upon reasonable request to the corresponding author.

## Ethics declaration

This study focuses on invertebrates that does not require review and/or approval by an ethics committee.

## CRediT authorship contribution statement

**Catarina F. Lourenço:** Writing – original draft, Methodology, Investigation. **Ana R. Almeida:** Writing – review & editing, Supervision, Methodology, Investigation. **Amadeu M.V.M. Soares:** Writing – review & editing, Resources, Investigation, Funding acquisition. **Catarina R. Marques:** Writing – review & editing, Supervision, Resources, Methodology, Investigation, Funding acquisition, Conceptualization.

## Declaration of competing interest

The authors declare that they have no known competing financial interests or personal relationships that could have appeared to influence the work reported in this paper.
