# Supplementary material for: Efficiency comparison of DNA extraction kits for analysing the cockle gut bacteriome
Source: Heliyon. 2024 Oct 9;10(20):e38846. doi: 10.1016/j.heliyon.2024.e38846 (PMC11620152; doi:10.1016/j.heliyon.2024.e38846)
Supplement: Multimedia component 1 [file mmc1.pdf]

Supplementary material

Efficiency Comparison of DNA Extraction Kits for Analysing the Cockle Gut Bacteriome

Catarina F. Lourenço, Ana R. Almeida, Amadeu M.V.M. Soares, Catarina R. Marques*

Center for Environmental and Marine Studies (CESAM) & Department of Biology, University of Aveiro, Campus Universitário de Santiago, 3810-193, Aveiro, Portugal

* E-mail address: crmarques@ua.pt

1. Supplementary Tables and Figures

1.1. Supplementary Tables

Table S1 - Major extraction steps instructed by the manufacturer for each of the five DNA extraction kits.

Extraction steps	E.Z.N.A.	FastDNA	DNeasy	PowerFecal	Zymo
**Sample preparation**	Sample added to glass beads; SLX-Mlus buffer added.	Sample added to Lysing Matrix A Tube.	Sample added to PowerBead Pro Tube; Solution CD1 added.	Sample added to PowerBead and C1 solutions.	Sample added to ZR BashingBead Lysis Tubes.
**Cell lysis**	Vortex-homogenised. DS buffer added; incubate 2 times (70 ºC and 95 ºC).	CLS-TC added; homogenised in FastPrep Instrutment.	Homogenised in vortex.	Heated at 65 ºC. Vortex-homogenised. Solution C2 added; incubated at 2-8 ºC.	ZymoBIOMICS lysis solution added. Vortex-homogenised.
**Inhibitor removal**	P2 buffer added; incubated at -20 ºC; isopropanol added; incubated at 70 ºC; HTR Reagent added.	-	Solution CD2 added.	Solution C3 added; incubated at 2-8ºC.	Supernatant transferred to Zymo-Spin III-F Filter.
**DNA Binding**	XP1 Buffer added; loaded onto HiBind DNA Mini Column.	Added to Binding Matrix.	CD3 solution added; loaded onto MB Spin Column.	Solution C4 added; loaded onto MB Spin Column.	ZymoBIOMICS DNA Binding Buffer added; mixture transferred to Zymo-Spin IICR Column.
**Wash**	Washed with XP1 Buffer and SPW Buffer.	Washed with SEWS-M.	Washed with Solution EA, and Solution C5.	Washed with Solution C5.	Add ZymoBIOMICS DNA Wash Buffer 1; ZymoBIOMICS DNA Wash Buffer 2 added.
**Elute DNA**	Eluted with Elution Buffer heated at 70 ºC; incubated at 70 ºC for 5'.	Eluted with DES; incubated at 55 ºC.	Eluted with Solution C6.	Eluted with Solution C6.	Eluted with ZymoBIOMICS DNase/ RNase Free Water; Zymo BIOMICS HCR Prep Solution added.

Table S2 – Summary of the one-way analysis of variance (*F*) for different parameters associated with the DNA extraction undertaken with the five kits. *[DNA]*- concentration of DNA; *df* – degrees of freedom; *P value* – value of probability.

**Parameter**	**Source of variation**	***df***	***F***	***P value***
[DNA]	Between Groups	4,19	30.508	<0.001
Amount of DNA / mg gut	Between Groups	4,19	24.996	<0.001
Extraction efficiency	Between Groups	4,19	12.012	<0.001

Table S3 – Summary of the one-way ANOVA applied to the DGGE band patterns obtained from the PCR amplicons for the five extraction kits.

DNA extraction kits	Source of variance	Degree of Freedom (*df*)	Sum square (*SS*)	Mean Square (*MS*)	*F* ratio	*P* value	*F critic*
E.Z.N.A.	Between replicates	0.000003	3	0.000001	0.000522	0.999983	2.669256
	Within replicates	0.226196	140	0.001616			
	Total	0.226198	143				
FastDNA	Between replicates	0.001373	4	0.000343	0.039056	0.997074	2.423286
	Within replicates	1.538241	175	0.008790			
	Total	1.539614	179				
PowerFecal	Between replicates	0.000014	4.0	0.000003	0.001499	0.999995	2.423286
	Within replicates	0.400576	175.0	0.002289			
	Total	0.400590	179.0				
Dneasy	Between replicates	0.000042	4	0.000010	0.004865	0.999952	2.423286
	Within replicates	0.374653	175	0.002141			
	Total	0.374695	179				
Zymo	Between replicates	0.000006	4	0.000001	0.000844	0.999999	2.423286
	Within replicates	0.309652	175	0.001769			
	Total	0.309658	179				

Table S4 – Relative abundance of bacterial OTUs identified in the *Cerastoderma edule* gut by four DNA extraction kits (E.Z.N.A., DNeasy, PowerFecal, and Zymo). nd – not determined.

#OTU ID	E.Z.N.A.	DNeasy	PowerFecal	Zymo	SUM	Phylum	Class	Order	Family	Genus	Species	Gram Stain
OTU_2	0.12893	0.10069	0.13987	0.16360	0.53309	Tenericutes	Mollicutes	Mycoplasmatales	Mycoplasmataceae	*Mycoplasma*	*M. marinum*	-
OTU_1	0.29687	0.06042	0.06400	0.03858	0.45987	Tenericutes	Mollicutes	Mycoplasmatales	Mycoplasmataceae	*Mycoplasma*	*M. marinum*	-
OTU_4	0.00316	0.19845	0.12507	0.07611	0.40278	Tenericutes	Mollicutes	Mycoplasmatales	Mycoplasmataceae	*Mycoplasma*	*M. marinum*	-
OTU_5	0.04512	0.05751	0.06976	0.13754	0.30992	Tenericutes	Mollicutes	Mycoplasmatales	Mycoplasmataceae	*Mycoplasma*	*M. marinum*	-
OTU_8	0.04500	0.02539	0.03203	0.04668	0.14910	Tenericutes	Mollicutes	Mycoplasmatales	Mycoplasmataceae	*Mycoplasma*	*M. marinum*	-
OTU_7	0.03904	0.03627	0.02036	0.05130	0.14697	Tenericutes	Mollicutes	Mycoplasmatales	Mycoplasmataceae	*Mycoplasma*	*M. marinum*	-
OTU_10	0.00721	0.03737	0.00631	0.09002	0.14090	Proteobacteria	Alphaproteobacteria	Rickettsiales	Anaplasmataceae	*Anaplasma*	*A. phagocytophilum*	-
OTU_3	0.01773	0.02615	0.02664	0.03997	0.11049	Proteobacteria	Epsilonproteobacteria	Campylobacterales	Campylobacteraceae	*Arcobacter*	*A. nitrofigilis*	-
OTU_12	0.00755	0.01975	0.02772	0.03397	0.08899	Tenericutes	Mollicutes	Mycoplasmatales	Mycoplasmataceae	*Mycoplasma*	*M. marinum*	-
OTU_6	0.05537	0.00805	0.00173	0.00095	0.06610	Proteobacteria	Gammaproteobacteria	Thiotrichales	Fastidiosibacteraceae	*Cysteiniphilum*	*nd*	-
OTU_14	0.01924	0.01527	0.00807	0.00952	0.05210	Tenericutes	Mollicutes	Mycoplasmatales	Mycoplasmataceae	*Mycoplasma*	*M. marinum*	-
OTU_15	0.00000	0.00422	0.03413	0.00750	0.04584	Proteobacteria	Deltaproteobacteria	Desulfobacterales	Desulfobulbaceae	*Desulfobulbus*	*nd*	-
OTU_9	0.00000	0.01404	0.01530	0.01612	0.04545	Proteobacteria	Gammaproteobacteria	Oceanospirillales	Endozoicomonadaceae	*Endozoicomonas*	*nd*	-
OTU_20	0.00000	0.04392	0.00000	0.00000	0.04392	Tenericutes	Mollicutes	Mycoplasmatales	Mycoplasmataceae	*Mycoplasma*	*M. marinum*	-
OTU_19	0.00239	0.00870	0.00843	0.02345	0.04298	Tenericutes	Mollicutes	Mycoplasmatales	Mycoplasmataceae	*Mycoplasma*	*M. marinum*	-
OTU_24	0.00000	0.02106	0.00700	0.01021	0.03826	Tenericutes	Mollicutes	Mycoplasmatales	Mycoplasmataceae	*Mycoplasma*	*M. marinum*	-
OTU_11	0.00000	0.02971	0.00000	0.00591	0.03562	Bacteroidetes	Bacteroidia	Marinilabiliales	Marinilabiliaceae	*Saccharicrinis*	*nd*	-
OTU_17	0.00000	0.01717	0.00425	0.01230	0.03372	Proteobacteria	Gammaproteobacteria	Chromatiales	Woeseiaceae	*Woeseia*	*W. oceani*	-
OTU_21	0.02747	0.00000	0.00000	0.00000	0.02747	Tenericutes	Mollicutes	Mycoplasmatales	Mycoplasmataceae	*Mycoplasma*	*M. marinum*	-
OTU_16	0.00163	0.00282	0.01772	0.00420	0.02636	Proteobacteria	Deltaproteobacteria	Desulfobacterales	Desulfobulbaceae	*Desulfopila*	*nd*	-
OTU_32	0.00000	0.00862	0.00291	0.01381	0.02535	Tenericutes	Mollicutes	Mycoplasmatales	Mycoplasmataceae	*Mycoplasma*	*M. marinum*	-
OTU_33	0.00804	0.00336	0.00454	0.00707	0.02301	Tenericutes	Mollicutes	Mycoplasmatales	Mycoplasmataceae	*Mycoplasma*	*M. marinum*	-
OTU_63	0.00000	0.00000	0.02282	0.00000	0.02282	Proteobacteria	Gammaproteobacteria	Chromatiales	Ectothiorhodospiraceae	*Thiohalomonas*	*T. nitratireducens*	-
OTU_46	0.00000	0.01354	0.00546	0.00372	0.02272	Tenericutes	Mollicutes	Mycoplasmatales	Mycoplasmataceae	*Mycoplasma*	*M. marinum*	-
OTU_30	0.00296	0.00000	0.00000	0.01954	0.02250	Tenericutes	Mollicutes	Mycoplasmatales	Mycoplasmataceae	*Mycoplasma*	*M. mobile*	+
OTU_27	0.01732	0.00258	0.00242	0.00000	0.02232	Tenericutes	Mollicutes	Mycoplasmatales	Mycoplasmataceae	*Mycoplasma*	*M. marinum*	-
OTU_23	0.00000	0.00195	0.00000	0.02028	0.02222	Chlamydiae	Chlamydiia	Parachlamydiales	Criblamydiaceae	*Criblamydia*	*C. sequanensis*	-
OTU_28	0.00000	0.00542	0.01661	0.00000	0.02202	Bacteroidetes	Flavobacteriia	Flavobacteriales	Flavobacteriaceae	*Tenacibaculum*	*nd*	-
OTU_36	0.00575	0.00408	0.00559	0.00629	0.02170	Tenericutes	Mollicutes	Mycoplasmatales	Mycoplasmataceae	*Mycoplasma*	*M. marinum*	-

OTU_51	0.00000	0.01214	0.00575	0.00364	0.02154	Tenericutes	Mollicutes	Mycoplasmatales	Mycoplasmataceae	*Mycoplasma*	*marinum*	-
OTU_40	0.00000	0.00241	0.01442	0.00321	0.02004	Actinobacteria	Acidimicrobiia	Acidimicrobiales	Ilumatobacteraceae	*Ilumatobacter*	*I. fluminis*	+
OTU_34	0.00000	0.01670	0.00000	0.00128	0.01798	Spirochaetes	Spirochaetia	Spirochaetales	Spirochaetaceae	*Spirochaeta*	*sp.BHI80-158*	-
OTU_37	0.01282	0.00212	0.00281	0.00000	0.01775	Tenericutes	Mollicutes	Mycoplasmatales	Mycoplasmataceae	*Mycoplasma*	*M. marinum*	-
OTU_31	0.00000	0.00249	0.01013	0.00411	0.01674	Bacteroidetes	Flavobacteriia	Flavobacteriales	Flavobacteriaceae	*Lutimonas*	*L. saemankumensis*	-
OTU_26	0.00000	0.00000	0.01461	0.00000	0.01461	Proteobacteria	Gammaproteobacteria	Vibrionales	Vibrionaceae	*Vibrio*	*nd*	-
OTU_49	0.00000	0.00103	0.00814	0.00542	0.01458	Proteobacteria	Gammaproteobacteria	Cellvibrionales	Halieaceae	*Luminiphilus*	*L. syltensis*	-
OTU_38	0.01426	0.00000	0.00000	0.00000	0.01426	Tenericutes	Mollicutes	Mycoplasmatales	Mycoplasmataceae	*Mycoplasma*	sp.VJC358	-
OTU_25	0.00000	0.01181	0.00000	0.00214	0.01396	Bacteroidetes	Bacteroidia	Marinilabiliales	Marinilabiliaceae	*Saccharicrinis*	*nd*	-
OTU_47	0.00000	0.00095	0.01049	0.00235	0.01379	Bacteroidetes	Flavobacteriia	Flavobacteriales	Flavobacteriaceae	*Actibacter*	*nd*	-
OTU_41	0.01372	0.00000	0.00000	0.00000	0.01372	Tenericutes	Mollicutes	Mycoplasmatales	Mycoplasmataceae	*Mycoplasma*	sp.VJC358	-
OTU_29	0.00000	0.00000	0.01366	0.00000	0.01366	Proteobacteria	Gammaproteobacteria	Vibrionales	Vibrionaceae	*Vibrio*	*nd*	-
OTU_45	0.00000	0.00135	0.00919	0.00263	0.01317	Bacteroidetes	Flavobacteriia	Flavobacteriales	Flavobacteriaceae	*Actibacter*	*nd*	-
OTU_58	0.00000	0.00342	0.00000	0.00911	0.01253	Tenericutes	Mollicutes	Mycoplasmatales	Mycoplasmataceae	*Mycoplasma*	*M. marinum*	-
OTU_62	0.00000	0.00000	0.00801	0.00428	0.01229	Proteobacteria	Gammaproteobacteria	Chromatiales	Wenzhouxiangellaceae	*Wenzhouxiangella*	*W. salilacus*	-
OTU_61	0.00150	0.00209	0.00255	0.00580	0.01193	Tenericutes	Mollicutes	Mycoplasmatales	Mycoplasmataceae	*Mycoplasma*	*M. marinum*	-
OTU_48	0.00211	0.00300	0.00494	0.00134	0.01139	Proteobacteria	Gammaproteobacteria	Thiotrichales	Fastidiosibacteraceae	*Fangia*	*F. hongkongensis*	-
OTU_13	0.00372	0.00400	0.00108	0.00220	0.01100	Proteobacteria	Gammaproteobacteria	Vibrionales	Vibrionaceae	*Vibrio*	*nd*	-
OTU_54	0.00000	0.00112	0.00716	0.00188	0.01016	Proteobacteria	Deltaproteobacteria	Desulfobacterales	Desulfobulbaceae	*Desulfopila*	*nd*	-
OTU_44	0.00107	0.00000	0.00605	0.00266	0.00977	Proteobacteria	Epsilonproteobacteria	Campylobacterales	Helicobacteraceae	*Sulfurovum*	*nd*	-
OTU_42	0.00386	0.00224	0.00216	0.00122	0.00948	Proteobacteria	Gammaproteobacteria	Thiotrichales	Fastidiosibacteraceae	*Cysteiniphilum*	*nd*	-
OTU_86	0.00000	0.00591	0.00000	0.00326	0.00918	Proteobacteria	Alphaproteobacteria	Rickettsiales	Rickettsiaceae	*Rickettsia*	*nd*	-
OTU_74	0.00293	0.00314	0.00000	0.00308	0.00915	Proteobacteria	Alphaproteobacteria	Rickettsiales	Rickettsiaceae	*Rickettsia*	*Endosymbiont of Bemisia tabaci*	-
OTU_133	0.00000	0.00000	0.00909	0.00000	0.00909	Proteobacteria	Gammaproteobacteria	Chromatiales	Ectothiorhodospiraceae	*nd*	*nd*	-
OTU_65	0.00000	0.00265	0.00454	0.00186	0.00905	Actinobacteria	Acidimicrobiia	Acidimicrobiales	Ilumatobacteraceae	*Ilumatobacter*	*I. nonamiensis*	+
OTU_35	0.00135	0.00199	0.00101	0.00451	0.00886	Bacteroidetes	Bacteroidia	Marinilabiliales	Marinilabiliaceae	*Labilibacter*	*L. marinus*	-
OTU_67	0.00000	0.00269	0.00209	0.00387	0.00865	Tenericutes	Mollicutes	Entomoplasmatales	Spiroplasmataceae	*Spiroplasma*	*S. platyhelix*	+
OTU_88	0.00000	0.00000	0.00297	0.00532	0.00829	Tenericutes	Mollicutes	Mycoplasmatales	Mycoplasmataceae	*Mycoplasma*	*M. marinum*	-
OTU_18	0.00818	0.00000	0.00000	0.00000	0.00818	Proteobacteria	Gammaproteobacteria	Pseudomonadales	Pseudomonadaceae	*Pseudomonas*	*nd*	-
OTU_43	0.00000	0.00414	0.00000	0.00384	0.00798	Proteobacteria	Epsilonproteobacteria	Campylobacterales	Campylobacteraceae	*Sulfurospirillum*	*S. carboxydovorans*	-
OTU_52	0.00000	0.00629	0.00000	0.00137	0.00765	Bacteroidetes	Bacteroidia	Marinilabiliales	Marinilabiliaceae	*Saccharicrinis*	*nd*	-
OTU_107	0.00000	0.00000	0.00346	0.00379	0.00726	Tenericutes	Mollicutes	Mycoplasmatales	Mycoplasmataceae	*Mycoplasma*	*M. marinum*	-
OTU_82	0.00000	0.00000	0.00438	0.00254	0.00692	Actinobacteria	Acidimicrobiia	Acidimicrobiales	Ilumatobacteraceae	*Ilumatobacter*	*I. fluminis*	+

OTU_57	0.00413	0.00000	0.00000	0.00228	0.00640	Bacteroidetes	Flavobacteriia	Flavobacteriales	Flavobacteriaceae	*Polaribacter*	*nd*	-
OTU_95	0.00000	0.00125	0.00000	0.00510	0.00634	Tenericutes	Mollicutes	Mycoplasmatales	Mycoplasmataceae	*Mycoplasma*	*M. marinum*	-
OTU_97	0.00000	0.00135	0.00000	0.00492	0.00628	Tenericutes	Mollicutes	Mycoplasmatales	Mycoplasmataceae	*Mycoplasma*	*M. marinum*	-
OTU_59	0.00000	0.00100	0.00526	0.00000	0.00626	Fusobacteria	Fusobacteriia	Fusobacteriales	Fusobacteriaceae	*Ilyobacter*	*I. psychrophilus*	-
OTU_22	0.00306	0.00195	0.00000	0.00118	0.00619	Proteobacteria	Gammaproteobacteria	Vibrionales	Vibrionaceae	*Vibrio*	*nd*	-
OTU_100	0.00000	0.00075	0.00536	0.00000	0.00611	Bacteroidetes	Flavobacteriia	Flavobacteriales	Weeksellaceae	*Spongiimonas*	*S. flava*	-
OTU_68	0.00000	0.00000	0.00386	0.00224	0.00610	Proteobacteria	Alphaproteobacteria	Rhodobacterales	Rhodobacteraceae	*Roseobacter*	*R. denitrificans*	-
OTU_94	0.00000	0.00000	0.00000	0.00609	0.00609	Tenericutes	Mollicutes	Mycoplasmatales	Mycoplasmataceae	*Mycoplasma*	*M. marinum*	-
OTU_99	0.00000	0.00000	0.00441	0.00166	0.00607	Proteobacteria	Gammaproteobacteria	Cellvibrionales	Halieaceae	*nd*	*nd*	-
OTU_39	0.00000	0.00378	0.00000	0.00225	0.00604	Proteobacteria	Gammaproteobacteria	Oceanospirillales	Endozoicomonadaceae	*Endozoicomonas*	*E. ascidiicola*	-
OTU_223	0.00000	0.00000	0.00588	0.00000	0.00588	Actinobacteria	Nitriliruptoria	Egibacterales	Egibacteraceae	*Egibacter*	*E. rhizosphaerae*	-
OTU_75	0.00583	0.00000	0.00000	0.00000	0.00583	Tenericutes	Mollicutes	Mycoplasmatales	Mycoplasmataceae	*Mycoplasma*	*M. marinum*	-
OTU_55	0.00000	0.00375	0.00000	0.00204	0.00579	Proteobacteria	Deltaproteobacteria	Desulfobacterales	Desulfobulbaceae	*CandidatusElectrothrix*	*C.E. japonica*	-
OTU_122	0.00000	0.00556	0.00000	0.00000	0.00556	Tenericutes	Mollicutes	Mycoplasmatales	Mycoplasmataceae	*Mycoplasma*	*M. marinum*	-
OTU_83	0.00544	0.00000	0.00000	0.00000	0.00544	Tenericutes	Mollicutes	Mycoplasmatales	Mycoplasmataceae	*Mycoplasma*	*M. marinum*	-
OTU_92	0.00000	0.00000	0.00441	0.00102	0.00543	Bacteroidetes	Flavobacteriia	Flavobacteriales	Flavobacteriaceae	*Lutimonas*	*L. vermicola*	-
OTU_160	0.00000	0.00097	0.00438	0.00000	0.00535	Proteobacteria	Alphaproteobacteria	Rhizobiales	Hyphomicrobiaceae	*Filomicrobium*	*nd*	-
OTU_84	0.00534	0.00000	0.00000	0.00000	0.00534	Tenericutes	Mollicutes	Mycoplasmatales	Mycoplasmataceae	*Mycoplasma*	*M. marinum*	-
OTU_128	0.00000	0.00520	0.00000	0.00000	0.00520	Tenericutes	Mollicutes	Mycoplasmatales	Mycoplasmataceae	*Mycoplasma*	*M. marinum*	-
OTU_66	0.00000	0.00000	0.00507	0.00000	0.00507	Proteobacteria	Gammaproteobacteria	Vibrionales	Vibrionaceae	*Vibrio*	*nd*	-
OTU_93	0.00494	0.00000	0.00000	0.00000	0.00494	Tenericutes	Mollicutes	Mycoplasmatales	Mycoplasmataceae	*Mycoplasma*	*M. marinum*	-
OTU_50	0.00000	0.00403	0.00000	0.00080	0.00483	Proteobacteria	Epsilonproteobacteria	Campylobacterales	Campylobacteraceae	*Arcobacter*	*nd*	-
OTU_116	0.00000	0.00000	0.00000	0.00481	0.00481	Tenericutes	Mollicutes	Mycoplasmatales	Mycoplasmataceae	*Mycoplasma*	*M. marinum*	-
OTU_139	0.00000	0.00475	0.00000	0.00000	0.00475	Tenericutes	Mollicutes	Mycoplasmatales	Mycoplasmataceae	*Mycoplasma*	*M. marinum*	-
OTU_130	0.00000	0.00000	0.00471	0.00000	0.00471	Proteobacteria	Alphaproteobacteria	Rhodobacterales	Rhodobacteraceae	*Roseobacter*	*R. denitrificans*	-
OTU_114	0.00168	0.00114	0.00000	0.00188	0.00470	Tenericutes	Mollicutes	Mycoplasmatales	Mycoplasmataceae	*Mycoplasma*	*M. marinum*	-
OTU_53	0.00000	0.00372	0.00000	0.00095	0.00467	Fusobacteria	Fusobacteriia	Fusobacteriales	Fusobacteriaceae	*Psychrilyobacter*	*nd*	-
OTU_132	0.00000	0.00000	0.00464	0.00000	0.00464	Proteobacteria	Alphaproteobacteria	Rhodobacterales	Rhodobacteraceae	*Roseobacter*	*R. sp. p28*	-
OTU_142	0.00000	0.00464	0.00000	0.00000	0.00464	Tenericutes	Mollicutes	Mycoplasmatales	Mycoplasmataceae	*Mycoplasma*	*M. marinum*	-
OTU_70	0.00000	0.00000	0.00461	0.00000	0.00461	Proteobacteria	Gammaproteobacteria	Vibrionales	Vibrionaceae	*Vibrio*	*nd*	-
OTU_101	0.00430	0.00000	0.00000	0.00000	0.00430	Tenericutes	Mollicutes	Mycoplasmatales	Mycoplasmataceae	*Mycoplasma*	*M. marinum*	-
OTU_103	0.00419	0.00000	0.00000	0.00000	0.00419	Tenericutes	Mollicutes	Mycoplasmatales	Mycoplasmataceae	*Mycoplasma*	*M. marinum*	-
OTU_104	0.00415	0.00000	0.00000	0.00000	0.00415	Tenericutes	Mollicutes	Mycoplasmatales	Mycoplasmataceae	*Mycoplasma*	*M. marinum*	-

OTU_106	0.00410	0.00000	0.00000	0.00000	0.00410	Tenericutes	Mollicutes	Mycoplasmatales	Mycoplasmataceae	*Mycoplasma*	*M. marinum*	-
OTU_108	0.00409	0.00000	0.00000	0.00000	0.00409	Tenericutes	Mollicutes	Mycoplasmatales	Mycoplasmataceae	*Mycoplasma*	*M. marinum*	-
OTU_193	0.00000	0.00000	0.00409	0.00000	0.00409	Proteobacteria	Gammaproteobacteria	Chromatiales	Thioalkalispiraceae	*Thiohalophilus*	*T. thiocyanatoxydans*	-
OTU_143	0.00000	0.00000	0.00409	0.00000	0.00409	Proteobacteria	Alphaproteobacteria	Rhodobacterales	Rhodobacteraceae	*Tateyamaria*	*nd*	-
OTU_76	0.00000	0.00000	0.00405	0.00000	0.00405	Proteobacteria	Gammaproteobacteria	Vibrionales	Vibrionaceae	*Vibrio*	*nd*	-
OTU_71	0.00000	0.00405	0.00000	0.00000	0.00405	Bacteroidetes	Bacteroidia	Marinilabiliales	Marinifilaceae	*Ancylomarina*	*A. subtilis*	-
OTU_120	0.00000	0.00000	0.00317	0.00079	0.00396	Bacteroidetes	Flavobacteriia	Flavobacteriales	Flavobacteriaceae	*Lutimonas*	*nd*	-
OTU_169	0.00000	0.00000	0.00392	0.00000	0.00392	Proteobacteria	Gammaproteobacteria	Cellvibrionales	Halieaceae	*Haliea*	*H. sp.SY02*	-
OTU_171	0.00000	0.00388	0.00000	0.00000	0.00388	Proteobacteria	Alphaproteobacteria	Rickettsiales	Rickettsiaceae	*CandidatusMegaira*	*C.M. sp.OL-2017*	-
OTU_60	0.00065	0.00097	0.00075	0.00143	0.00380	Proteobacteria	Epsilonproteobacteria	Campylobacterales	Campylobacteraceae	*Arcobacter*	*A. nitrofigilis*	-
OTU_118	0.00374	0.00000	0.00000	0.00000	0.00374	Tenericutes	Mollicutes	Mycoplasmatales	Mycoplasmataceae	*Mycoplasma*	*M. sp.VJC358*	-
OTU_78	0.00000	0.00370	0.00000	0.00000	0.00370	Bacteroidetes	Bacteroidia	Marinilabiliales	Marinifilaceae	*Ancylomarina*	*A. salipaludis*	-
OTU_72	0.00000	0.00367	0.00000	0.00000	0.00367	Proteobacteria	Deltaproteobacteria	Desulfovibrionales	Desulfovibrionaceae	*Desulfovibrio*	*D. sp.La1.3*	-
OTU_205	0.00000	0.00000	0.00363	0.00000	0.00363	Proteobacteria	Gammaproteobacteria	Chromatiales	Chromatiaceae	*nd*	*nd*	-
OTU_81	0.00000	0.00223	0.00000	0.00138	0.00360	Firmicutes	Clostridia	Clostridiales	Lachnospiraceae	*Niameybacter*	*N. massiliensis*	+
OTU_157	0.00000	0.00352	0.00000	0.00000	0.00352	Tenericutes	Mollicutes	Mycoplasmatales	Mycoplasmataceae	*Mycoplasma*	*M. marinum*	-
OTU_123	0.00346	0.00000	0.00000	0.00000	0.00346	Tenericutes	Mollicutes	Mycoplasmatales	Mycoplasmataceae	*Mycoplasma*	*M. marinum*	-
OTU_79	0.00000	0.00257	0.00000	0.00086	0.00343	Bacteroidetes	Bacteroidia	Bacteroidales	Bacteroidaceae	*Bacteroides*	*B. graminisolvens*	-
OTU_77	0.00000	0.00000	0.00144	0.00196	0.00340	Proteobacteria	Deltaproteobacteria	Desulfobacterales	Desulfobacteraceae	*nd*	*nd*	-
OTU_126	0.00334	0.00000	0.00000	0.00000	0.00334	Tenericutes	Mollicutes	Mycoplasmatales	Mycoplasmataceae	*Mycoplasma*	*M. marinum*	-
OTU_147	0.00000	0.00000	0.00000	0.00326	0.00326	Tenericutes	Mollicutes	Mycoplasmatales	Mycoplasmataceae	*Mycoplasma*	*M. marinum*	-
OTU_131	0.00320	0.00000	0.00000	0.00000	0.00320	Tenericutes	Mollicutes	Mycoplasmatales	Mycoplasmataceae	*Mycoplasma*	*M. marinum*	-
OTU_170	0.00000	0.00319	0.00000	0.00000	0.00319	Tenericutes	Mollicutes	Mycoplasmatales	Mycoplasmataceae	*Mycoplasma*	*M. marinum*	-
OTU_134	0.00318	0.00000	0.00000	0.00000	0.00318	Tenericutes	Mollicutes	Mycoplasmatales	Mycoplasmataceae	*Mycoplasma*	*M. marinum*	-
OTU_113	0.00000	0.00182	0.00000	0.00135	0.00318	Actinobacteria	Actinobacteria	Geodermatophilales	Geodermatophilaceae	*Modestobacter*	*M. versicolor*	+
OTU_102	0.00000	0.00314	0.00000	0.00000	0.00314	Bacteroidetes	Cytophagia	Cytophagales	Cytophagaceae	*Cytophaga*	*C. sp.N05VI*	-
OTU_135	0.00314	0.00000	0.00000	0.00000	0.00314	Tenericutes	Mollicutes	Mycoplasmatales	Mycoplasmataceae	*Mycoplasma*	*M. marinum*	-
OTU_243	0.00000	0.00000	0.00314	0.00000	0.00314	Proteobacteria	Gammaproteobacteria	Chromatiales	*nd*	*nd*	*nd*	-
OTU_136	0.00312	0.00000	0.00000	0.00000	0.00312	Tenericutes	Mollicutes	Mycoplasmatales	Mycoplasmataceae	*Mycoplasma*	*M. marinum*	-
OTU_221	0.00000	0.00000	0.00301	0.00000	0.00301	Actinobacteria	Acidimicrobiia	Acidimicrobiales	Ilumatobacteraceae	*Ilumatobacter*	*I. nonamiensis*	+
OTU_91	0.00292	0.00000	0.00000	0.00000	0.00292	Proteobacteria	Gammaproteobacteria	Enterobacterales	Yersiniaceae	*Serratia*	*nd*	-
OTU_69	0.00292	0.00000	0.00000	0.00000	0.00292	Proteobacteria	Oligoflexia	Silvanigrellales	Silvanigrellaceae	*Silvanigrella*	*S. aquatica*	-
OTU_98	0.00000	0.00285	0.00000	0.00000	0.00285	Proteobacteria	Deltaproteobacteria	Desulfovibrionales	Desulfovibrionaceae	*Desulfovibrio*	*D. sp.La1.3*	-

OTU_80	0.00000	0.00097	0.00088	0.00100	0.00284	Proteobacteria	Gammaproteobacteria	Oceanospirillales	Endozoicomonadaceae	*Endozoicomonas*	*nd*	-
OTU_229	0.00000	0.00000	0.00284	0.00000	0.00284	Actinobacteria	Acidimicrobiia	Acidimicrobiales	Ilumatobacteraceae	*Ilumatobacter*	*I. fluminis*	+
OTU_224	0.00000	0.00000	0.00281	0.00000	0.00281	Proteobacteria	Gammaproteobacteria	*nd*	*nd*	*Thiolapillus*	*T. brandeum*	-
OTU_232	0.00000	0.00000	0.00271	0.00000	0.00271	Proteobacteria	Gammaproteobacteria	Cellvibrionales	Halieaceae	*Marimicrobium*	*M. arenosum*	-
OTU_200	0.00000	0.00269	0.00000	0.00000	0.00269	Tenericutes	Mollicutes	Mycoplasmatales	Mycoplasmataceae	*Mycoplasma*	*M. marinum*	-
OTU_295	0.00000	0.00000	0.00268	0.00000	0.00268	Proteobacteria	Gammaproteobacteria	Chromatiales	Ectothiorhodospiraceae	*Inmirania*	*I. thermothiophila*	-
OTU_183	0.00000	0.00000	0.00265	0.00000	0.00265	Bacteroidetes	Flavobacteriia	Flavobacteriales	Flavobacteriaceae	*nd*	*nd*	-
OTU_241	0.00000	0.00000	0.00262	0.00000	0.00262	Proteobacteria	Gammaproteobacteria	Cellvibrionales	Halieaceae	*Halioglobus*	*H. sediminis*	-
OTU_148	0.00261	0.00000	0.00000	0.00000	0.00261	Tenericutes	Mollicutes	Mycoplasmatales	Mycoplasmataceae	*Mycoplasma*	*M. marinum*	-
OTU_275	0.00000	0.00000	0.00255	0.00000	0.00255	Proteobacteria	Gammaproteobacteria	Chromatiales	*nd*	*nd*	*nd*	-
OTU_313	0.00000	0.00000	0.00252	0.00000	0.00252	Acidobacteria	Thermoanaerobaculia	Thermoanaerobaculales	Thermoanaerobaculaceae	*Thermoanaerobaculum*	*T. aquaticum*	-
OTU_172	0.00000	0.00000	0.00252	0.00000	0.00252	Proteobacteria	Epsilonproteobacteria	Campylobacterales	Helicobacteraceae	*Sulfurovum*	*nd*	-
OTU_173	0.00000	0.00000	0.00000	0.00251	0.00251	Tenericutes	Mollicutes	Mycoplasmatales	Mycoplasmataceae	*Mycoplasma*	*M. marinum*	-
OTU_271	0.00000	0.00000	0.00248	0.00000	0.00248	Proteobacteria	Gammaproteobacteria	Chromatiales	Chromatiaceae	*Halochromatium*	*H. glycolicum*	-
OTU_250	0.00000	0.00000	0.00248	0.00000	0.00248	Proteobacteria	Gammaproteobacteria	Cellvibrionales	Halieaceae	*nd*	*nd*	-
OTU_236	0.00000	0.00000	0.00245	0.00000	0.00245	Proteobacteria	Alphaproteobacteria	Rhizobiales	Rhodobiaceae	*Anderseniella*	*A. baltica*	-
OTU_198	0.00000	0.00000	0.00245	0.00000	0.00245	Bacteroidetes	Flavobacteriia	Flavobacteriales	Flavobacteriaceae	*Actibacter*	*nd*	-
OTU_272	0.00000	0.00000	0.00245	0.00000	0.00245	Proteobacteria	Gammaproteobacteria	Chromatiales	Chromatiaceae	*Thiohalocapsa*	*T. halophila*	-
OTU_176	0.00000	0.00000	0.00000	0.00244	0.00244	Tenericutes	Mollicutes	Mycoplasmatales	Mycoplasmataceae	*Mycoplasma*	*M. marinum*	-
OTU_73	0.00000	0.00087	0.00000	0.00156	0.00244	Proteobacteria	Gammaproteobacteria	Oceanospirillales	Endozoicomonadaceae	*Kistimonas*	*K. sp.1804*	-
OTU_276	0.00000	0.00000	0.00242	0.00000	0.00242	Proteobacteria	Gammaproteobacteria	Chromatiales	Chromatiaceae	*Halochromatium*	*glycolicum*	-
OTU_216	0.00000	0.00241	0.00000	0.00000	0.00241	Tenericutes	Mollicutes	Mycoplasmatales	Mycoplasmataceae	*Mycoplasma*	*M. marinum*	-
OTU_56	0.00164	0.00076	0.00000	0.00000	0.00240	Proteobacteria	Gammaproteobacteria	Vibrionales	Vibrionaceae	*Vibrio*	*nd*	-
OTU_211	0.00000	0.00000	0.00000	0.00239	0.00239	Proteobacteria	Alphaproteobacteria	Rickettsiales	Anaplasmataceae	*Anaplasma*	*A. phagocytophilum*	-
OTU_282	0.00000	0.00000	0.00239	0.00000	0.00239	Proteobacteria	Gammaproteobacteria	Chromatiales	Wenzhouxiangellaceae	*Wenzhouxiangella*	*nd*	-
OTU_129	0.00000	0.00000	0.00000	0.00236	0.00236	Proteobacteria	Gammaproteobacteria	Cellvibrionales	Microbulbiferaceae	*Microbulbifer*	*M. okinawensis*	-
OTU_305	0.00000	0.00000	0.00235	0.00000	0.00235	Proteobacteria	Gammaproteobacteria	Chromatiales	Ectothiorhodospiraceae	*Thiohalomonas*	*T. nitratireducens*	-
OTU_256	0.00000	0.00235	0.00000	0.00000	0.00235	Proteobacteria	Alphaproteobacteria	Rickettsiales	Rickettsiaceae	*Rickettsia*	*nd*	-
OTU_85	0.00230	0.00000	0.00000	0.00000	0.00230	Bacteroidetes	Flavobacteriia	Flavobacteriales	Flavobacteriaceae	*Lutibacter*	*L. holmesii*	-
OTU_155	0.00229	0.00000	0.00000	0.00000	0.00229	Tenericutes	Mollicutes	Mycoplasmatales	Mycoplasmataceae	*Mycoplasma*	*M. marinum*	-
OTU_213	0.00000	0.00000	0.00229	0.00000	0.00229	Proteobacteria	Alphaproteobacteria	Rhodobacterales	Rhodobacteraceae	*Marivita*	*M. sp.AC122-G30*	-
OTU_87	0.00228	0.00000	0.00000	0.00000	0.00228	Proteobacteria	Alphaproteobacteria	Rhodobacterales	Rhodobacteraceae	*nd*	*nd*	-
OTU_186	0.00000	0.00000	0.00000	0.00225	0.00225	Tenericutes	Mollicutes	Mycoplasmatales	Mycoplasmataceae	*Mycoplasma*	*M. marinum*	-

OTU_156	0.00225	0.00000	0.00000	0.00000	0.00225	Tenericutes	Mollicutes	Mycoplasmatales	Mycoplasmataceae	*Mycoplasma*	*M. marinum*	-
OTU_178	0.00000	0.00000	0.00222	0.00000	0.00222	Proteobacteria	Deltaproteobacteria	Desulfobacterales	Desulfobacteraceae	*Desulfosarcina*	*D. alkanivorans*	-
OTU_89	0.00222	0.00000	0.00000	0.00000	0.00222	Bacteroidetes	Flavobacteriia	Flavobacteriales	Flavobacteriaceae	*Tenacibaculum*	*T. sp.MED341*	-
OTU_231	0.00000	0.00221	0.00000	0.00000	0.00221	Tenericutes	Mollicutes	Mycoplasmatales	Mycoplasmataceae	*Mycoplasma*	*M. marinum*	-
OTU_125	0.00117	0.00000	0.00000	0.00103	0.00221	Proteobacteria	Gammaproteobacteria	Cellvibrionales	Halieaceae	*nd*	*nd*	-
OTU_325	0.00000	0.00000	0.00216	0.00000	0.00216	Tenericutes	Mollicutes	Mycoplasmatales	Mycoplasmataceae	*Mycoplasma*	*M. marinum*	-
OTU_162	0.00215	0.00000	0.00000	0.00000	0.00215	Tenericutes	Mollicutes	Mycoplasmatales	Mycoplasmataceae	*Mycoplasma*	*M. marinum*	-
OTU_222	0.00000	0.00000	0.00212	0.00000	0.00212	Bacteroidetes	Flavobacteriia	Flavobacteriales	Flavobacteriaceae	*Lutimonas*	*L. sp.MOLA107*	-
OTU_105	0.00129	0.00000	0.00000	0.00080	0.00209	Bacteroidetes	*nd*	*nd*	*nd*	*nd*	*nd*	-
OTU_298	0.00000	0.00000	0.00209	0.00000	0.00209	Proteobacteria	Gammaproteobacteria	Chromatiales	Woeseiaceae	*Woeseia*	*W. oceani*	-
OTU_163	0.00207	0.00000	0.00000	0.00000	0.00207	Tenericutes	Mollicutes	Mycoplasmatales	Mycoplasmataceae	*Mycoplasma*	*M. marinum*	-
OTU_164	0.00207	0.00000	0.00000	0.00000	0.00207	Tenericutes	Mollicutes	Mycoplasmatales	Mycoplasmataceae	*Mycoplasma*	*M. marinum*	-
OTU_165	0.00206	0.00000	0.00000	0.00000	0.00206	Tenericutes	Mollicutes	Mycoplasmatales	Mycoplasmataceae	*Mycoplasma*	*M. marinum*	-
OTU_227	0.00000	0.00000	0.00206	0.00000	0.00206	Bacteroidetes	Flavobacteriia	Flavobacteriales	Flavobacteriaceae	*Lutimonas*	*L. sp.KHS05*	-
OTU_279	0.00000	0.00000	0.00206	0.00000	0.00206	Proteobacteria	Gammaproteobacteria	Arenicellales	Arenicellaceae	*Arenicella*	*nd*	-
OTU_109	0.00127	0.00000	0.00000	0.00078	0.00205	Bacteroidetes	*nd*	*nd*	*nd*	*nd*	*nd*	-
OTU_167	0.00204	0.00000	0.00000	0.00000	0.00204	Tenericutes	Mollicutes	Mycoplasmatales	Mycoplasmataceae	*Mycoplasma*	*M. marinum*	-
OTU_203	0.00000	0.00000	0.00203	0.00000	0.00203	Proteobacteria	Epsilonproteobacteria	Campylobacterales	Helicobacteraceae	*Sulfurovum*	*nd*	-
OTU_168	0.00202	0.00000	0.00000	0.00000	0.00202	Tenericutes	Mollicutes	Mycoplasmatales	Mycoplasmataceae	*Mycoplasma*	*M. marinum*	-
OTU_299	0.00000	0.00000	0.00199	0.00000	0.00199	Proteobacteria	Gammaproteobacteria	Chromatiales	Chromatiaceae	*nd*	*nd*	-
OTU_253	0.00000	0.00199	0.00000	0.00000	0.00199	Tenericutes	Mollicutes	Mycoplasmatales	Mycoplasmataceae	*Mycoplasma*	*M. marinum*	-
OTU_294	0.00000	0.00000	0.00196	0.00000	0.00196	Proteobacteria	Deltaproteobacteria	Desulfobacterales	Desulfobulbaceae	*Desulfobulbus*	*nd*	-
OTU_303	0.00000	0.00000	0.00196	0.00000	0.00196	Proteobacteria	Gammaproteobacteria	Chromatiales	Wenzhouxiangellaceae	*Wenzhouxiangella*	*nd*	-
OTU_310	0.00000	0.00000	0.00186	0.00000	0.00186	Proteobacteria	Gammaproteobacteria	Chromatiales	Wenzhouxiangellaceae	*Wenzhouxiangella*	*nd*	-
OTU_290	0.00000	0.00000	0.00186	0.00000	0.00186	Actinobacteria	Actinobacteria	Streptomycetales	Streptomycetaceae	*Streptomyces*	*S. aomiensis*	+
OTU_140	0.00000	0.00185	0.00000	0.00000	0.00185	Proteobacteria	Deltaproteobacteria	Desulfobacterales	Desulfobulbaceae	*Desulfopila*	*D. sp.PR8_E11*	-
OTU_182	0.00185	0.00000	0.00000	0.00000	0.00185	Tenericutes	Mollicutes	Mycoplasmatales	Mycoplasmataceae	*Mycoplasma*	*M. sp.VJC358*	-
OTU_296	0.00000	0.00000	0.00183	0.00000	0.00183	Proteobacteria	Gammaproteobacteria	Cellvibrionales	Halieaceae	*nd*	*nd*	-
OTU_254	0.00000	0.00000	0.00183	0.00000	0.00183	Proteobacteria	Alphaproteobacteria	Rhodobacterales	Rhodobacteraceae	*Pseudoruegeria*	*nd*	-
OTU_292	0.00000	0.00000	0.00180	0.00000	0.00180	Proteobacteria	Alphaproteobacteria	*nd*	*nd*	*nd*	*nd*	+
OTU_297	0.00000	0.00000	0.00180	0.00000	0.00180	Actinobacteria	Acidimicrobiia	Acidimicrobiales	Ilumatobacteraceae	*Ilumatobacter*	*I. nonamiensis*	-
OTU_188	0.00177	0.00000	0.00000	0.00000	0.00177	Tenericutes	Mollicutes	Mycoplasmatales	Mycoplasmataceae	*Mycoplasma*	*M. marinum*	-
OTU_189	0.00177	0.00000	0.00000	0.00000	0.00177	Tenericutes	Mollicutes	Mycoplasmatales	Mycoplasmataceae	*Mycoplasma*	*M. marinum*	-

OTU_261	0.00000	0.00000	0.00177	0.00000	0.00177	Proteobacteria	Alphaproteobacteria	Rhodobacterales	Rhodobacteraceae	*Sulfitobacter*	*nd*	-
OTU_190	0.00176	0.00000	0.00000	0.00000	0.00176	Tenericutes	Mollicutes	Mycoplasmatales	Mycoplasmataceae	*Mycoplasma*	*M. marinum*	-
OTU_138	0.00175	0.00000	0.00000	0.00000	0.00175	Proteobacteria	Alphaproteobacteria	Sphingomonadales	Sphingomonadaceae	*Zymomonas*	*nd*	-
OTU_192	0.00174	0.00000	0.00000	0.00000	0.00174	Tenericutes	Mollicutes	Mycoplasmatales	Mycoplasmataceae	*Mycoplasma*	*M. marinum*	-
OTU_195	0.00173	0.00000	0.00000	0.00000	0.00173	Tenericutes	Mollicutes	Mycoplasmatales	Mycoplasmataceae	*Mycoplasma*	*M. marinum*	-
OTU_194	0.00173	0.00000	0.00000	0.00000	0.00173	Tenericutes	Mollicutes	Mycoplasmatales	Mycoplasmataceae	*Mycoplasma*	*M. marinum*	-
OTU_199	0.00171	0.00000	0.00000	0.00000	0.00171	Tenericutes	Mollicutes	Mycoplasmatales	Mycoplasmataceae	*Mycoplasma*	*M. marinum*	-
OTU_280	0.00000	0.00168	0.00000	0.00000	0.00168	Tenericutes	Mollicutes	Mycoplasmatales	Mycoplasmataceae	*Mycoplasma*	*M. marinum*	-
OTU_202	0.00163	0.00000	0.00000	0.00000	0.00163	Tenericutes	Mollicutes	Mycoplasmatales	Mycoplasmataceae	*Mycoplasma*	*M. marinum*	-
OTU_244	0.00000	0.00000	0.00000	0.00163	0.00163	Tenericutes	Mollicutes	Mycoplasmatales	Mycoplasmataceae	*Mycoplasma*	*M. marinum*	-
OTU_204	0.00162	0.00000	0.00000	0.00000	0.00162	Tenericutes	Mollicutes	Mycoplasmatales	Mycoplasmataceae	*Mycoplasma*	*M. marinum*	-
OTU_206	0.00161	0.00000	0.00000	0.00000	0.00161	Tenericutes	Mollicutes	Mycoplasmatales	Mycoplasmataceae	*Mycoplasma*	*M. marinum*	-
OTU_209	0.00159	0.00000	0.00000	0.00000	0.00159	Tenericutes	Mollicutes	Mycoplasmatales	Mycoplasmataceae	*Mycoplasma*	*M. marinum*	-
OTU_285	0.00000	0.00159	0.00000	0.00000	0.00159	Tenericutes	Mollicutes	Mycoplasmatales	Mycoplasmataceae	*Mycoplasma*	*M. marinum*	-
OTU_274	0.00000	0.00000	0.00157	0.00000	0.00157	Bacteroidetes	Flavobacteriia	Flavobacteriales	Flavobacteriaceae	*Marinirhabdus*	*M. citrea*	-
OTU_214	0.00155	0.00000	0.00000	0.00000	0.00155	Tenericutes	Mollicutes	Mycoplasmatales	Mycoplasmataceae	*Mycoplasma*	*M. marinum*	-
OTU_215	0.00155	0.00000	0.00000	0.00000	0.00155	Tenericutes	Mollicutes	Mycoplasmatales	Mycoplasmataceae	*Mycoplasma*	*M. marinum*	-
OTU_127	0.00154	0.00000	0.00000	0.00000	0.00154	Bacteroidetes	Cytophagia	Cytophagales	Flammeovirgaceae	*Sediminitomix*	*S. flava*	-
OTU_111	0.00000	0.00000	0.00000	0.00154	0.00154	Proteobacteria	Epsilonproteobacteria	Campylobacterales	Campylobacteraceae	*Arcobacter*	*A. nitrofigilis*	-
OTU_278	0.00000	0.00000	0.00154	0.00000	0.00154	Bacteroidetes	Flavobacteriia	Flavobacteriales	Flavobacteriaceae	*Actibacter*	*nd*	-
OTU_119	0.00000	0.00106	0.00000	0.00047	0.00153	Firmicutes	Clostridia	Eubacteriales	Lachnospiraceae	*Natranaerovirga*	*N. pectinivora*	+
OTU_149	0.00152	0.00000	0.00000	0.00000	0.00152	Proteobacteria	Gammaproteobacteria	*nd*	*nd*	*nd*	*nd*	-
OTU_217	0.00151	0.00000	0.00000	0.00000	0.00151	Tenericutes	Mollicutes	Mycoplasmatales	Mycoplasmataceae	*Mycoplasma*	*M. marinum*	-
OTU_219	0.00149	0.00000	0.00000	0.00000	0.00149	Tenericutes	Mollicutes	Mycoplasmatales	Mycoplasmataceae	*Mycoplasma*	*M. marinum*	-
OTU_218	0.00149	0.00000	0.00000	0.00000	0.00149	Tenericutes	Mollicutes	Mycoplasmatales	Mycoplasmataceae	*Mycoplasma*	*M. marinum*	-
OTU_220	0.00148	0.00000	0.00000	0.00000	0.00148	Tenericutes	Mollicutes	Mycoplasmatales	Mycoplasmataceae	*Mycoplasma*	*M. marinum*	-
OTU_314	0.00000	0.00000	0.00147	0.00000	0.00147	Actinobacteria	Acidimicrobiia	Acidimicrobiales	Ilumatobacteraceae	*Ilumatobacter*	*I. nonamiensis*	+
OTU_226	0.00144	0.00000	0.00000	0.00000	0.00144	Tenericutes	Mollicutes	Mycoplasmatales	Mycoplasmataceae	*Mycoplasma*	*M. marinum*	-
OTU_225	0.00144	0.00000	0.00000	0.00000	0.00144	Tenericutes	Mollicutes	Mycoplasmatales	Mycoplasmataceae	*Mycoplasma*	*M. marinum*	-
OTU_288	0.00000	0.00000	0.00144	0.00000	0.00144	Proteobacteria	Alphaproteobacteria	Rhodobacterales	Rhodobacteraceae	*nd*	*nd*	-
OTU_283	0.00000	0.00000	0.00144	0.00000	0.00144	Bacteroidetes	Flavobacteriia	Flavobacteriales	Flavobacteriaceae	*Taeania*	*T. maliponensis*	-
OTU_289	0.00000	0.00000	0.00144	0.00000	0.00144	Proteobacteria	Alphaproteobacteria	Rhodobacterales	Rhodobacteraceae	*Pseudoruegeria*	*P. insulae*	-
OTU_228	0.00141	0.00000	0.00000	0.00000	0.00141	Tenericutes	Mollicutes	Mycoplasmatales	Mycoplasmataceae	*Mycoplasma*	*M. marinum*	-

OTU_152	0.00140	0.00000	0.00000	0.00000	0.00140	Proteobacteria	Gammaproteobacteria	Enterobacterales	Yersiniaceae	*Serratia*	*nd*	-
OTU_230	0.00140	0.00000	0.00000	0.00000	0.00140	Tenericutes	Mollicutes	Mycoplasmatales	Mycoplasmataceae	*Mycoplasma*	*M. marinum*	-
OTU_233	0.00139	0.00000	0.00000	0.00000	0.00139	Tenericutes	Mollicutes	Mycoplasmatales	Mycoplasmataceae	*Mycoplasma*	*M. marinum*	-
OTU_235	0.00139	0.00000	0.00000	0.00000	0.00139	Tenericutes	Mollicutes	Mycoplasmatales	Mycoplasmataceae	*Mycoplasma*	*M. marinum*	-
OTU_264	0.00000	0.00000	0.00137	0.00000	0.00137	Proteobacteria	Deltaproteobacteria	Desulfobacterales	Desulfobacteraceae	*Desulfococcus*	*nd*	-
OTU_237	0.00137	0.00000	0.00000	0.00000	0.00137	Tenericutes	Mollicutes	Mycoplasmatales	Mycoplasmataceae	*Mycoplasma*	*M. marinum*	-
OTU_64	0.00137	0.00000	0.00000	0.00000	0.00137	Proteobacteria	Gammaproteobacteria	Vibrionales	Vibrionaceae	*nd*	*nd*	-
OTU_238	0.00135	0.00000	0.00000	0.00000	0.00135	Tenericutes	Mollicutes	Mycoplasmatales	Mycoplasmataceae	*Mycoplasma*	*M. marinum*	-
OTU_239	0.00135	0.00000	0.00000	0.00000	0.00135	Tenericutes	Mollicutes	Mycoplasmatales	Mycoplasmataceae	*Mycoplasma*	*M. marinum*	-
OTU_240	0.00134	0.00000	0.00000	0.00000	0.00134	Tenericutes	Mollicutes	Mycoplasmatales	Mycoplasmataceae	*Mycoplasma*	*M. marinum*	-
OTU_293	0.00000	0.00000	0.00131	0.00000	0.00131	Proteobacteria	Alphaproteobacteria	Rhodospirillales	Geminicoccaceae	*CandidatusCombothrix*	*italica*	-
OTU_304	0.00000	0.00131	0.00000	0.00000	0.00131	Tenericutes	Mollicutes	Mycoplasmatales	Mycoplasmataceae	*Mycoplasma*	*M. marinum*	-
OTU_247	0.00130	0.00000	0.00000	0.00000	0.00130	Tenericutes	Mollicutes	Mycoplasmatales	Mycoplasmataceae	*Mycoplasma*	*M. marinum*	-
OTU_246	0.00130	0.00000	0.00000	0.00000	0.00130	Tenericutes	Mollicutes	Mycoplasmatales	Mycoplasmataceae	*Mycoplasma*	*M. marinum*	-
OTU_245	0.00130	0.00000	0.00000	0.00000	0.00130	Tenericutes	Mollicutes	Mycoplasmatales	Mycoplasmataceae	*Mycoplasma*	*M. marinum*	-
OTU_141	0.00000	0.00129	0.00000	0.00000	0.00129	Firmicutes	Clostridia	Clostridiales	*nd*	*Acidaminobacter*	*nd*	-
OTU_306	0.00000	0.00129	0.00000	0.00000	0.00129	Tenericutes	Mollicutes	Mycoplasmatales	Mycoplasmataceae	*Mycoplasma*	*M. marinum*	-
OTU_307	0.00000	0.00128	0.00000	0.00000	0.00128	Tenericutes	Mollicutes	Mycoplasmatales	Mycoplasmataceae	*Mycoplasma*	*M. marinum*	-
OTU_308	0.00000	0.00128	0.00000	0.00000	0.00128	Tenericutes	Mollicutes	Mycoplasmatales	Mycoplasmataceae	*Mycoplasma*	*M. marinum*	-
OTU_251	0.00127	0.00000	0.00000	0.00000	0.00127	Tenericutes	Mollicutes	Mycoplasmatales	Mycoplasmataceae	*Mycoplasma*	*M. marinum*	-
OTU_249	0.00127	0.00000	0.00000	0.00000	0.00127	Tenericutes	Mollicutes	Mycoplasmatales	Mycoplasmataceae	*Mycoplasma*	*M. marinum*	-
OTU_252	0.00126	0.00000	0.00000	0.00000	0.00126	Tenericutes	Mollicutes	Mycoplasmatales	Mycoplasmataceae	*Mycoplasma*	*M. marinum*	-
OTU_137	0.00000	0.00126	0.00000	0.00000	0.00126	Proteobacteria	Epsilonproteobacteria	Campylobacterales	Campylobacteraceae	*Arcobacter*	*nd*	-
OTU_166	0.00000	0.00126	0.00000	0.00000	0.00126	Proteobacteria	Deltaproteobacteria	Desulfobacterales	Desulfobulbaceae	*CandidatusElectrothrix*	*C.E. japonica*	-
OTU_255	0.00123	0.00000	0.00000	0.00000	0.00123	Tenericutes	Mollicutes	Mycoplasmatales	Mycoplasmataceae	*Mycoplasma*	*M. marinum*	-
OTU_309	0.00000	0.00123	0.00000	0.00000	0.00123	Tenericutes	Mollicutes	Mycoplasmatales	Mycoplasmataceae	*Mycoplasma*	*M. marinum*	-
OTU_174	0.00000	0.00123	0.00000	0.00000	0.00123	Proteobacteria	Deltaproteobacteria	Desulfobacterales	Desulfobulbaceae	*CandidatusElectrothrix*	*C.E. japonica*	-
OTU_257	0.00122	0.00000	0.00000	0.00000	0.00122	Tenericutes	Mollicutes	Mycoplasmatales	Mycoplasmataceae	*Mycoplasma*	*M. marinum*	-
OTU_201	0.00000	0.00000	0.00000	0.00122	0.00122	Proteobacteria	Gammaproteobacteria	Cellvibrionales	Halieaceae	*Haliea*	*H. sp.ETY-M*	-
OTU_260	0.00121	0.00000	0.00000	0.00000	0.00121	Tenericutes	Mollicutes	Mycoplasmatales	Mycoplasmataceae	*Mycoplasma*	*M. marinum*	-
OTU_259	0.00121	0.00000	0.00000	0.00000	0.00121	Tenericutes	Mollicutes	Mycoplasmatales	Mycoplasmataceae	*Mycoplasma*	*M. marinum*	-
OTU_258	0.00121	0.00000	0.00000	0.00000	0.00121	Tenericutes	Mollicutes	Mycoplasmatales	Mycoplasmataceae	*Mycoplasma*	*M. marinum*	-
OTU_311	0.00000	0.00121	0.00000	0.00000	0.00121	Tenericutes	Mollicutes	Mycoplasmatales	Mycoplasmataceae	*Mycoplasma*	*M. marinum*	-

OTU_121	0.00000	0.00121	0.00000	0.00000	0.00121	Proteobacteria	Gammaproteobacteria	Vibrionales	Vibrionaceae	*Vibrio*	*V. sp.r32*	-
OTU_263	0.00120	0.00000	0.00000	0.00000	0.00120	Tenericutes	Mollicutes	Mycoplasmatales	Mycoplasmataceae	*Mycoplasma*	*M. marinum*	-
OTU_262	0.00120	0.00000	0.00000	0.00000	0.00120	Tenericutes	Mollicutes	Mycoplasmatales	Mycoplasmataceae	*Mycoplasma*	*M. marinum*	-
OTU_146	0.00119	0.00000	0.00000	0.00000	0.00119	Bacteroidetes	Flavobacteriia	Flavobacteriales	Flavobacteriaceae	*Lutibacter*	*L. holmesii*	-
OTU_175	0.00000	0.00118	0.00000	0.00000	0.00118	Proteobacteria	Deltaproteobacteria	Desulfovibrionales	Desulfovibrionaceae	*Desulfovibrio*	*nd*	-
OTU_284	0.00000	0.00000	0.00118	0.00000	0.00118	Proteobacteria	Deltaproteobacteria	Desulfobacterales	Desulfobacteraceae	*Desulfococcus*	*nd*	-
OTU_177	0.00117	0.00000	0.00000	0.00000	0.00117	Bacteroidetes	Flavobacteriia	Flavobacteriales	Flavobacteriaceae	*Polaribacter*	*nd*	-
OTU_312	0.00000	0.00117	0.00000	0.00000	0.00117	Tenericutes	Mollicutes	Mycoplasmatales	Mycoplasmataceae	*Mycoplasma*	*M. marinum*	-
OTU_265	0.00116	0.00000	0.00000	0.00000	0.00116	Tenericutes	Mollicutes	Mycoplasmatales	Mycoplasmataceae	*Mycoplasma*	*M. marinum*	-
OTU_302	0.00000	0.00000	0.00114	0.00000	0.00114	Proteobacteria	Alphaproteobacteria	Rhodospirillales	Geminicoccaceae	*CandidatusCombothrix*	*C.C. italica*	-
OTU_315	0.00000	0.00114	0.00000	0.00000	0.00114	Tenericutes	Mollicutes	Mycoplasmatales	Mycoplasmataceae	*Mycoplasma*	*M. marinum*	-
OTU_159	0.00113	0.00000	0.00000	0.00000	0.00113	*nd*	*nd*	*nd*	*nd*	*nd*	*nd*	
OTU_210	0.00000	0.00000	0.00000	0.00112	0.00112	Proteobacteria	Alphaproteobacteria	Sphingomonadales	Sphingomonadaceae	*Zymomonas*	*nd*	-
OTU_337	0.00000	0.00000	0.00111	0.00000	0.00111	Actinobacteria	Acidimicrobiia	Acidimicrobiales	Ilumatobacteraceae	*Ilumatobacter*	*I. nonamiensis*	+
OTU_270	0.00111	0.00000	0.00000	0.00000	0.00111	Tenericutes	Mollicutes	Mycoplasmatales	Mycoplasmataceae	*Mycoplasma*	*M. marinum*	-
OTU_269	0.00111	0.00000	0.00000	0.00000	0.00111	Tenericutes	Mollicutes	Mycoplasmatales	Mycoplasmataceae	*Mycoplasma*	*M. marinum*	-
OTU_316	0.00000	0.00111	0.00000	0.00000	0.00111	Tenericutes	Mollicutes	Mycoplasmatales	Mycoplasmataceae	*Mycoplasma*	*M. marinum*	-
OTU_317	0.00000	0.00111	0.00000	0.00000	0.00111	Tenericutes	Mollicutes	Mycoplasmatales	Mycoplasmataceae	*Mycoplasma*	*M. marinum*	-
OTU_318	0.00000	0.00109	0.00000	0.00000	0.00109	Tenericutes	Mollicutes	Mycoplasmatales	Mycoplasmataceae	*Mycoplasma*	*M. marinum*	-
OTU_320	0.00000	0.00107	0.00000	0.00000	0.00107	Tenericutes	Mollicutes	Mycoplasmatales	Mycoplasmataceae	*Mycoplasma*	*M. marinum*	-
OTU_90	0.00107	0.00000	0.00000	0.00000	0.00107	Proteobacteria	Gammaproteobacteria	Vibrionales	Vibrionaceae	*Vibrio*	*nd*	-
OTU_277	0.00107	0.00000	0.00000	0.00000	0.00107	Tenericutes	Mollicutes	Mycoplasmatales	Mycoplasmataceae	*Mycoplasma*	*M. marinum*	-
OTU_144	0.00000	0.00000	0.00000	0.00106	0.00106	Proteobacteria	Epsilonproteobacteria	Campylobacterales	Campylobacteraceae	*Arcobacter*	*A. nitrofigilis*	-
OTU_321	0.00000	0.00106	0.00000	0.00000	0.00106	Tenericutes	Mollicutes	Mycoplasmatales	Mycoplasmataceae	*Mycoplasma*	*M. marinum*	-
OTU_281	0.00105	0.00000	0.00000	0.00000	0.00105	Tenericutes	Mollicutes	Mycoplasmatales	Mycoplasmataceae	*Mycoplasma*	*M. marinum*	-
OTU_323	0.00000	0.00104	0.00000	0.00000	0.00104	Tenericutes	Mollicutes	Mycoplasmatales	Mycoplasmataceae	*Mycoplasma*	*M. marinum*	-
OTU_324	0.00000	0.00104	0.00000	0.00000	0.00104	Tenericutes	Mollicutes	Mycoplasmatales	Mycoplasmataceae	*Mycoplasma*	*M. marinum*	-
OTU_150	0.00000	0.00103	0.00000	0.00000	0.00103	Proteobacteria	Epsilonproteobacteria	Campylobacterales	Campylobacteraceae	*Arcobacter*	*nd*	-
OTU_181	0.00000	0.00000	0.00000	0.00100	0.00100	Bacteroidetes	Flavobacteriia	Flavobacteriales	Flavobacteriaceae	*nd*	*nd*	-
OTU_326	0.00000	0.00098	0.00000	0.00000	0.00098	Tenericutes	Mollicutes	Mycoplasmatales	Mycoplasmataceae	*Mycoplasma*	*M. marinum*	-
OTU_291	0.00098	0.00000	0.00000	0.00000	0.00098	Tenericutes	Mollicutes	Mycoplasmatales	Mycoplasmataceae	*Mycoplasma*	*M. marinum*	-
OTU_327	0.00000	0.00097	0.00000	0.00000	0.00097	Tenericutes	Mollicutes	Mycoplasmatales	Mycoplasmataceae	*Mycoplasma*	*M. marinum*	-
OTU_328	0.00000	0.00095	0.00000	0.00000	0.00095	Tenericutes	Mollicutes	Mycoplasmatales	Mycoplasmataceae	*Mycoplasma*	*M. marinum*	-

OTU_329	0.00000	0.00095	0.00000	0.00000	0.00095	Tenericutes	Mollicutes	Mycoplasmatales	Mycoplasmataceae	*Mycoplasma*	*M. marinum*	-
OTU_153	0.00095	0.00000	0.00000	0.00000	0.00095	Fibrobacteres	Chitinivibrionia	Chitinivibrionales	Chitinivibrionaceae	*Chitinivibrio*	*C. alkaliphilus*	-
OTU_331	0.00000	0.00093	0.00000	0.00000	0.00093	Tenericutes	Mollicutes	Mycoplasmatales	Mycoplasmataceae	*Mycoplasma*	*M. marinum*	-
OTU_334	0.00000	0.00093	0.00000	0.00000	0.00093	Tenericutes	Mollicutes	Mycoplasmatales	Mycoplasmataceae	*Mycoplasma*	*M. marinum*	-
OTU_332	0.00000	0.00093	0.00000	0.00000	0.00093	Tenericutes	Mollicutes	Mycoplasmatales	Mycoplasmataceae	*Mycoplasma*	*M. marinum*	-
OTU_330	0.00000	0.00093	0.00000	0.00000	0.00093	Tenericutes	Mollicutes	Mycoplasmatales	Mycoplasmataceae	*Mycoplasma*	*M. marinum*	-
OTU_158	0.00093	0.00000	0.00000	0.00000	0.00093	Bacteroidetes	Bacteroidia	Marinilabiliales	Prolixibacteraceae	*Prolixibacter*	*nd*	-
OTU_300	0.00088	0.00000	0.00000	0.00000	0.00088	Tenericutes	Mollicutes	Mycoplasmatales	Mycoplasmataceae	*Mycoplasma*	*M. marinum*	-
OTU_301	0.00087	0.00000	0.00000	0.00000	0.00087	Tenericutes	Mollicutes	Mycoplasmatales	Mycoplasmataceae	*Mycoplasma*	*M. marinum*	-
OTU_110	0.00087	0.00000	0.00000	0.00000	0.00087	Proteobacteria	Gammaproteobacteria	Vibrionales	Vibrionaceae	*Vibrio*	*nd*	-
OTU_112	0.00086	0.00000	0.00000	0.00000	0.00086	Proteobacteria	Gammaproteobacteria	Vibrionales	Vibrionaceae	*Vibrio*	*nd*	-
OTU_335	0.00000	0.00000	0.00085	0.00000	0.00085	Proteobacteria	Alphaproteobacteria	Rhodobacterales	Rhodobacteraceae	*Oceanicella*	*O. actignis*	-
OTU_207	0.00000	0.00084	0.00000	0.00000	0.00084	Firmicutes	Bacilli	Lactobacillales	Leuconostocaceae	*Leuconostoc*	*nd*	+
OTU_115	0.00083	0.00000	0.00000	0.00000	0.00083	Proteobacteria	Gammaproteobacteria	Vibrionales	Vibrionaceae	*Vibrio*	*nd*	-
OTU_179	0.00082	0.00000	0.00000	0.00000	0.00082	Bacteroidetes	Flavobacteriia	Flavobacteriales	Flavobacteriaceae	*nd*	*nd*	-
OTU_319	0.00000	0.00000	0.00082	0.00000	0.00082	Proteobacteria	Deltaproteobacteria	Desulfobacterales	Desulfobacteraceae	*nd*	*nd*	-
OTU_117	0.00080	0.00000	0.00000	0.00000	0.00080	Proteobacteria	Gammaproteobacteria	Vibrionales	Vibrionaceae	*Vibrio*	*V. sp.S2895*	-
OTU_187	0.00078	0.00000	0.00000	0.00000	0.00078	Bacteroidetes	Flavobacteriia	Flavobacteriales	Flavobacteriaceae	*Formosa*	*F. sp.Hel3_A1_48*	-
OTU_124	0.00073	0.00000	0.00000	0.00000	0.00073	Proteobacteria	Gammaproteobacteria	Vibrionales	Vibrionaceae	*Vibrio*	*nd*	-
OTU_322	0.00000	0.00000	0.00072	0.00000	0.00072	Firmicutes	Bacilli	Lactobacillales	Leuconostocaceae	*Leuconostoc*	*nd*	+
OTU_273	0.00000	0.00072	0.00000	0.00000	0.00072	Bacteroidetes	Bacteroidia	Marinilabiliales	Prolixibacteraceae	*Prolixibacter*	*nd*	-
OTU_96	0.00071	0.00000	0.00000	0.00000	0.00071	Firmicutes	Bacilli	Bacillales	Bacillaceae	*Bacillus*	*B. sp.R48_27f*	+
OTU_333	0.00000	0.00000	0.00069	0.00000	0.00069	Proteobacteria	Deltaproteobacteria	Desulfobacterales	Desulfobacteraceae	*CandidatusMagnetomorum*	*C.M. sp.1-7*	-
OTU_336	0.00000	0.00000	0.00065	0.00000	0.00065	Proteobacteria	Deltaproteobacteria	Desulfobacterales	Desulfobacteraceae	*CandidatusMagnetomorum*	*C.M. sp.1-7*	-
OTU_212	0.00000	0.00064	0.00000	0.00000	0.00064	Proteobacteria	Epsilonproteobacteria	Campylobacterales	Campylobacteraceae	*Arcobacter*	*nd*	-
OTU_242	0.00000	0.00062	0.00000	0.00000	0.00062	Proteobacteria	Gammaproteobacteria	Oceanospirillales	Endozoicomonadaceae	*Kistimonas*	*nd*	-
OTU_145	0.00059	0.00000	0.00000	0.00000	0.00059	Proteobacteria	Gammaproteobacteria	Vibrionales	Vibrionaceae	*Vibrio*	*nd*	-
OTU_196	0.00000	0.00059	0.00000	0.00000	0.00059	Proteobacteria	Gammaproteobacteria	Vibrionales	Vibrionaceae	*Vibrio*	*nd*	-
OTU_161	0.00000	0.00058	0.00000	0.00000	0.00058	Firmicutes	Clostridia	Clostridiales	Peptostreptococcaceae	*Paeniclostridium*	*P. ghonii*	+
OTU_151	0.00053	0.00000	0.00000	0.00000	0.00053	Proteobacteria	Gammaproteobacteria	Vibrionales	Vibrionaceae	*Vibrio*	*nd*	-
OTU_154	0.00000	0.00048	0.00000	0.00000	0.00048	Proteobacteria	Gammaproteobacteria	Alteromonadales	Shewanellaceae	*Shewanella*	*nd*	-
OTU_191	0.00000	0.00000	0.00000	0.00047	0.00047	Proteobacteria	Gammaproteobacteria	Vibrionales	Vibrionaceae	*Vibrio*	*nd*	-
OTU_234	0.00000	0.00047	0.00000	0.00000	0.00047	Proteobacteria	Gammaproteobacteria	Vibrionales	Vibrionaceae	*Vibrio*	*V. scophthalmi*	-

OTU_287	0.00000	0.00040	0.00000	0.00000	0.00040	Proteobacteria	Epsilonproteobacteria	Campylobacterales	Campylobacteraceae	*Arcobacter*	*nd*	-
OTU_184	0.00039	0.00000	0.00000	0.00000	0.00039	Proteobacteria	Gammaproteobacteria	Vibrionales	Vibrionaceae	*Vibrio*	*nd*	-
OTU_248	0.00000	0.00000	0.00000	0.00039	0.00039	Proteobacteria	Gammaproteobacteria	Oceanospirillales	Hahellaceae	*Zooshikella*	*nd*	-
OTU_267	0.00000	0.00039	0.00000	0.00000	0.00039	Proteobacteria	Gammaproteobacteria	Vibrionales	Vibrionaceae	*Vibrio*	*nd*	-
OTU_268	0.00000	0.00039	0.00000	0.00000	0.00039	Proteobacteria	Gammaproteobacteria	Vibrionales	Vibrionaceae	*Vibrio*	*nd*	-
OTU_266	0.00000	0.00039	0.00000	0.00000	0.00039	Proteobacteria	Gammaproteobacteria	Vibrionales	Vibrionaceae	*Vibrio*	*nd*	-
OTU_286	0.00000	0.00000	0.00000	0.00038	0.00038	Proteobacteria	Epsilonproteobacteria	Campylobacterales	Campylobacteraceae	*Arcobacter*	*A. nitrofigilis*	-
OTU_197	0.00037	0.00000	0.00000	0.00000	0.00037	Proteobacteria	Gammaproteobacteria	Vibrionales	Vibrionaceae	*Vibrio*	*nd*	-
OTU_208	0.00035	0.00000	0.00000	0.00000	0.00035	Proteobacteria	Gammaproteobacteria	Vibrionales	Vibrionaceae	*Vibrio*	*nd*	-
OTU_180	0.00025	0.00000	0.00000	0.00000	0.00025	Proteobacteria	Gammaproteobacteria	Alteromonadales	Shewanellaceae	*Shewanella*	*S. baltica*	-
OTU_185	0.00025	0.00000	0.00000	0.00000	0.00025	Proteobacteria	Gammaproteobacteria	Alteromonadales	Colwelliaceae	*Thalassomonas*	*nd*	-

Table S5 – Relative abundance of 21 bacterial operational taxonomic units (OTUs) identified in the *Cerastoderma edule* gut that are shared by the four DNA extraction kits (E.Z.N.A., DNeasy, PowerFecal, and Zymo).

#OTU ID	E.Z.N.A.	DNeasy	PowerFecal	Zymo	SUM	% [a]	Phylum	Class	Order	Family	Genus
OTU_2	0.12893	0.10069	0.13987	0.16360	0.53309	13.33	Tenericutes	Mollicutes	Mycoplasmatales	Mycoplasmataceae	*Mycoplasma*
OTU_1	0.29687	0.06042	0.06400	0.03858	0.45987	11.50	Tenericutes	Mollicutes	Mycoplasmatales	Mycoplasmataceae	*Mycoplasma*
OTU_4	0.00316	0.19845	0.12507	0.07611	0.40278	10.07	Tenericutes	Mollicutes	Mycoplasmatales	Mycoplasmataceae	*Mycoplasma*
OTU_5	0.04512	0.05751	0.06976	0.13754	0.30992	7.75	Tenericutes	Mollicutes	Mycoplasmatales	Mycoplasmataceae	*Mycoplasma*
OTU_8	0.04500	0.02539	0.03203	0.04668	0.14910	3.73	Tenericutes	Mollicutes	Mycoplasmatales	Mycoplasmataceae	*Mycoplasma*
OTU_7	0.03904	0.03627	0.02036	0.05130	0.14697	3.67	Tenericutes	Mollicutes	Mycoplasmatales	Mycoplasmataceae	*Mycoplasma*
OTU_10	0.00721	0.03737	0.00631	0.09002	0.14090	3.52	Proteobacteria	Alphaproteobacteria	Rickettsiales	Anaplasmataceae	*Anaplasma*
OTU_3	0.01773	0.02615	0.02664	0.03997	0.11049	2.76	Proteobacteria	Epsilonproteobacteria	Campylobacterales	Campylobacteraceae	*Arcobacter*
OTU_12	0.00755	0.01975	0.02772	0.03397	0.08899	2.22	Tenericutes	Mollicutes	Mycoplasmatales	Mycoplasmataceae	*Mycoplasma*
OTU_6	0.05537	0.00805	0.00173	0.00095	0.06610	1.65	Proteobacteria	Gammaproteobacteria	Thiotrichales	Fastidiosibacteraceae	*Cysteiniphilum*
OTU_14	0.01924	0.01527	0.00807	0.00952	0.05210	1.30	Tenericutes	Mollicutes	Mycoplasmatales	Mycoplasmataceae	*Mycoplasma*
OTU_19	0.00239	0.00870	0.00843	0.02345	0.04298	1.07	Tenericutes	Mollicutes	Mycoplasmatales	Mycoplasmataceae	*Mycoplasma*
OTU_16	0.00163	0.00282	0.01772	0.00420	0.02636	0.66	Proteobacteria	Deltaproteobacteria	Desulfobacterales	Desulfobulbaceae	*Desulfopila*
OTU_33	0.00804	0.00336	0.00454	0.00707	0.02301	0.58	Tenericutes	Mollicutes	Mycoplasmatales	Mycoplasmataceae	*Mycoplasma*
OTU_36	0.00575	0.00408	0.00559	0.00629	0.02170	0.54	Tenericutes	Mollicutes	Mycoplasmatales	Mycoplasmataceae	*Mycoplasma*
OTU_61	0.00150	0.00209	0.00255	0.00580	0.01193	0.30	Tenericutes	Mollicutes	Mycoplasmatales	Mycoplasmataceae	*Mycoplasma*

OTU_48	0.00211	0.00300	0.00494	0.00134	0.01139	0.28	Proteobacteria	Gammaproteobacteria	Thiotrichales	Fastidiosibacteraceae	*Fangia*
OTU_13	0.00372	0.00400	0.00108	0.00220	0.01100	0.28	Proteobacteria	Gammaproteobacteria	Vibrionales	Vibrionaceae	*Vibrio*
OTU_42	0.00386	0.00224	0.00216	0.00122	0.00948	0.24	Proteobacteria	Gammaproteobacteria	Thiotrichales	Fastidiosibacteraceae	*Cysteiniphilum*
OTU_35	0.00135	0.00199	0.00101	0.00451	0.00886	0.22	Bacteroidota	Bacteroidia	Marinilabiliales	Marinilabiliaceae	*Labilibacter*
OTU_60	0.00065	0.00097	0.00075	0.00143	0.00380	0.09	Proteobacteria	Epsilonproteobacteria	Campylobacterales	Campylobacteraceae	*Arcobacter*
		**SUM**				2.63086					
		[a] **Percent of the total relative abundance of entire community**				65.77					

Table S6 – List of operational taxonomic units (OTUs) affiliated to Gram-positive bacteria (being presented the respective Phylum, Class, Order, Family, Genus and Species) extracted from the *Cerastoderma edule* gut by four DNA extraction kits (E.Z.N.A., DNeasy, PowerFecal, and Zymo). nd – not determined.

#OTU ID	E.Z.N.A.	DNeasy	PowerFecal	Zymo	Phylum	Class	Order	Family	Genus	Species
OTU_30	x			x	Tenericutes	Mollicutes	Mycoplasmatales	Mycoplasmataceae	*Mycoplasma*	*M. mobile*
OTU_40		x	x	x	Actinobacteria	Acidimicrobiia	Acidimicrobiales	Ilumatobacteraceae	*Ilumatobacter*	*I. fluminis*
OTU_65		x	x	x	Actinobacteria	Acidimicrobiia	Acidimicrobiales	Ilumatobacteraceae	*Ilumatobacter*	*I. nonamiensis*
OTU_67		x	x	x	Tenericutes	Mollicutes	Entomoplasmatales	Spiroplasmataceae	*Spiroplasma*	*S. platyhelix*
OTU_82			x	x	Actinobacteria	Acidimicrobiia	Acidimicrobiales	Ilumatobacteraceae	*Ilumatobacter*	*I. fluminis*
OTU_81		x		x	Firmicutes	Clostridia	Clostridiales	Lachnospiraceae	*Niameybacter*	*N. massiliensis*
OTU_113		x		x	Actinobacteria	Actinobacteria	Geodermatophilales	Geodermatophilaceae	*Modestobacter*	*M. versicolor*
OTU_221			x		Actinobacteria	Acidimicrobiia	Acidimicrobiales	Ilumatobacteraceae	*Ilumatobacter*	*I. nonamiensis*
OTU_229			x		Actinobacteria	Acidimicrobiia	Acidimicrobiales	Ilumatobacteraceae	*Ilumatobacter*	*I. fluminis*
OTU_290			x		Actinobacteria	Actinobacteria	Streptomycetales	Streptomycetaceae	*Streptomyces*	*S. aomiensis*
OTU_297			x		Actinobacteria	Acidimicrobiia	Acidimicrobiales	Ilumatobacteraceae	*Ilumatobacter*	*I. nonamiensis*
OTU_119		x		x	Firmicutes	Clostridia	Clostridiales	nd	*Natranaerovirga*	*N. pectinivora*
OTU_314			x		Actinobacteria	Acidimicrobiia	Acidimicrobiales	Ilumatobacteraceae	*Ilumatobacter*	*I. nonamiensis*
OTU_337			x		Actinobacteria	Acidimicrobiia	Acidimicrobiales	Ilumatobacteraceae	*Ilumatobacter*	*I. nonamiensis*
OTU_207		x			Firmicutes	Bacilli	Lactobacillales	Leuconostocaceae	*Leuconostoc*	nd
OTU_322			x		Firmicutes	Bacilli	Lactobacillales	Leuconostocaceae	*Leuconostoc*	nd
OTU_96	x				Firmicutes	Bacilli	Bacillales	Bacillaceae	*Bacillus*	sp. R48_27f
OTU_161		x			Firmicutes	Clostridia	Clostridiales	Peptostreptococcaceae	*Paeniclostridium*	*P. ghonii*
**Total**	**2**	**8**	**11**	**8**						
**%**	**1%**	**2%**	**3%**	**2%**						

Table S7 – Relative abundance of the bacterial genera associated with *Cerastoderma edule* gut, as recovered by four DNA extraction kits (E.Z.N.A., DNeasy, PowerFecal, and Zymo). nd - not determined.

E.Z.N.A.	DNeasy	PowerFecal	Zymo	SUM	Phylum	Class	Order	Family	Genus
0.84583	0.70754	0.54295	0.70394	2.80026	Tenericutes	Mollicutes	Mycoplasmatales	Mycoplasmataceae	*Mycoplasma*
0.00721	0.03737	0.00631	0.09241	0.14329	Proteobacteria	Alphaproteobacteria	Rickettsiales	Anaplasmataceae	*Anaplasma*
0.01838	0.03448	0.02739	0.04518	0.12543	Proteobacteria	Epsilonproteobacteria	Campylobacterales	Campylobacteraceae	*Arcobacter*
0.05923	0.01029	0.00389	0.00217	0.07558	Proteobacteria	Gammaproteobacteria	Thiotrichales	Fastidiosibacteraceae	*Cysteiniphilum*
0.01581	0.01015	0.04308	0.00385	0.07289	Proteobacteria	Gammaproteobacteria	Vibrionales	Vibrionaceae	*Vibrio*
0.00000	0.04781	0.00000	0.00942	0.05723	Bacteroidetes	Bacteroidia	Marinilabiliales	Marinilabiliaceae	*Saccharicrinis*
0.00000	0.01879	0.01618	0.01937	0.05433	Proteobacteria	Gammaproteobacteria	Oceanospirillales	Endozoicomonadaceae	*Endozoicomonas*
0.00000	0.00422	0.03609	0.00750	0.04780	Proteobacteria	Deltaproteobacteria	Desulfobacterales	Desulfobulbaceae	*Desulfobulbus*
0.00000	0.00506	0.03357	0.00761	0.04624	Actinobacteria	Acidimicrobiia	Acidimicrobiales	Ilumatobacteraceae	*Ilumatobacter*
0.00163	0.00579	0.02488	0.00608	0.03838	Proteobacteria	Deltaproteobacteria	Desulfobacterales	Desulfobulbaceae	*Desulfopila*
0.00000	0.01717	0.00634	0.01230	0.03581	Proteobacteria	Gammaproteobacteria	Chromatiales	Woeseiaceae	*Woeseia*
0.00000	0.00230	0.02367	0.00499	0.03096	Bacteroidetes	Flavobacteriia	Flavobacteriales	Flavobacteriaceae	*Actibacter*
0.00000	0.00249	0.02190	0.00592	0.03031	Bacteroidetes	Flavobacteriia	Flavobacteriales	Flavobacteriaceae	*Lutimonas*
0.00000	0.00000	0.02517	0.00000	0.02517	Proteobacteria	Gammaproteobacteria	Chromatiales	Ectothiorhodospiraceae	*Thiohalomonas*
0.00222	0.00542	0.01661	0.00000	0.02424	Bacteroidetes	Flavobacteriia	Flavobacteriales	Flavobacteriaceae	*Tenacibaculum*
0.00000	0.00195	0.00000	0.02028	0.02222	Chlamydiae	Chlamydiia	Parachlamydiales	Criblamydiaceae	*Criblamydia*
0.00293	0.01141	0.00000	0.00634	0.02068	Proteobacteria	Alphaproteobacteria	Rickettsiales	Rickettsiaceae	*Rickettsia*
0.00000	0.00000	0.01422	0.00428	0.01850	Proteobacteria	Gammaproteobacteria	Chromatiales	Wenzhouxiangellaceae	*Wenzhouxiangella*
0.00000	0.01670	0.00000	0.00128	0.01798	Spirochaetes	Spirochaetia	Spirochaetales	Spirochaetaceae	*Spirochaeta*
0.00000	0.00000	0.01321	0.00224	0.01545	Proteobacteria	Alphaproteobacteria	Rhodobacterales	Rhodobacteraceae	*Roseobacter*
0.00000	0.00103	0.00814	0.00542	0.01458	Proteobacteria	Gammaproteobacteria	Cellvibrionales	Halieaceae	*Luminiphilus*
0.00107	0.00000	0.01059	0.00266	0.01432	Proteobacteria	Epsilonproteobacteria	Campylobacterales	Helicobacteraceae	*Sulfurovum*
0.00117	0.00000	0.00873	0.00270	0.01260	Proteobacteria	Gammaproteobacteria	Cellvibrionales	Halieaceae	nd
0.00211	0.00300	0.00494	0.00134	0.01139	Proteobacteria	Gammaproteobacteria	Thiotrichales	Fastidiosibacteraceae	*Fangia*
0.00000	0.00000	0.00909	0.00000	0.00909	Proteobacteria	Gammaproteobacteria	Chromatiales	Ectothiorhodospiraceae	nd
0.00135	0.00199	0.00101	0.00451	0.00886	Bacteroidetes	Bacteroidia	Marinilabiliales	Marinilabiliaceae	*Labilibacter*
0.00000	0.00269	0.00209	0.00387	0.00865	Tenericutes	Mollicutes	Entomoplasmatales	Spiroplasmataceae	*Spiroplasma*
0.00000	0.00624	0.00000	0.00204	0.00829	Proteobacteria	Deltaproteobacteria	Desulfobacterales	Desulfobulbaceae	*Candidatus Electrothrix*
0.00818	0.00000	0.00000	0.00000	0.00818	Proteobacteria	Gammaproteobacteria	Pseudomonadales	Pseudomonadaceae	*Pseudomonas*

0.00000	0.00414	0.00000	0.00384	0.00798	Proteobacteria	Epsilonproteobacteria	Campylobacterales	Campylobacteraceae	*Sulfurospirillum*
0.00000	0.00775	0.00000	0.00000	0.00775	Bacteroidetes	Bacteroidia	Marinilabiliales	Marinifilaceae	*Ancylomarina*
0.00000	0.00770	0.00000	0.00000	0.00770	Proteobacteria	Deltaproteobacteria	Desulfovibrionales	Desulfovibrionaceae	*Desulfovibrio*
0.00530	0.00000	0.00000	0.00228	0.00758	Bacteroidetes	Flavobacteriia	Flavobacteriales	Flavobacteriaceae	*Polaribacter*
0.00000	0.00100	0.00526	0.00000	0.00626	Fusobacteria	Fusobacteriia	Fusobacteriales	Fusobacteriaceae	*Ilyobacter*
0.00000	0.00075	0.00536	0.00000	0.00611	Bacteroidetes	Flavobacteriia	Flavobacteriales	Weeksellaceae	*Spongiimonas*
0.00000	0.00000	0.00588	0.00000	0.00588	Actinobacteria	Nitriliruptoria	Egibacterales	Egibacteraceae	*Egibacter*
0.00000	0.00000	0.00569	0.00000	0.00569	Proteobacteria	Gammaproteobacteria	Chromatiales	nd	nd
0.00000	0.00000	0.00562	0.00000	0.00562	Proteobacteria	Gammaproteobacteria	Chromatiales	Chromatiaceae	nd
0.00000	0.00097	0.00438	0.00000	0.00535	Proteobacteria	Alphaproteobacteria	Rhizobiales	Hyphomicrobiaceae	*Filomicrobium*
0.00000	0.00000	0.00392	0.00122	0.00514	Proteobacteria	Gammaproteobacteria	Cellvibrionales	Halieaceae	*Haliea*
0.00000	0.00000	0.00490	0.00000	0.00490	Proteobacteria	Gammaproteobacteria	Chromatiales	Chromatiaceae	*Halochromatium*
0.00000	0.00372	0.00000	0.00095	0.00467	Fusobacteria	Fusobacteriia	Fusobacteriales	Fusobacteriaceae	*Psychrilyobacter*
0.00082	0.00000	0.00265	0.00100	0.00446	Bacteroidetes	Flavobacteriia	Flavobacteriales	Flavobacteriaceae	nd
0.00432	0.00000	0.00000	0.00000	0.00432	Proteobacteria	Gammaproteobacteria	Enterobacterales	Yersiniaceae	*Serratia*
0.00000	0.00000	0.00226	0.00196	0.00421	Proteobacteria	Deltaproteobacteria	Desulfobacterales	Desulfobacteraceae	nd
0.00257	0.00000	0.00000	0.00158	0.00414	Bacteroidetes	nd	nd	nd	nd
0.00000	0.00000	0.00409	0.00000	0.00409	Proteobacteria	Alphaproteobacteria	Rhodobacterales	Rhodobacteraceae	*Tateyamaria*
0.00000	0.00000	0.00409	0.00000	0.00409	Proteobacteria	Gammaproteobacteria	Chromatiales	Thioalkalispiraceae	*Thiohalophilus*
0.00000	0.00388	0.00000	0.00000	0.00388	Proteobacteria	Alphaproteobacteria	Rickettsiales	Rickettsiaceae	*Candidatus Megaira*
0.00228	0.00000	0.00144	0.00000	0.00372	Proteobacteria	Alphaproteobacteria	Rhodobacterales	Rhodobacteraceae	nd
0.00000	0.00223	0.00000	0.00138	0.00360	Firmicutes	Clostridia	Clostridiales	Lachnospiraceae	*Niameybacter*
0.00349	0.00000	0.00000	0.00000	0.00349	Bacteroidetes	Flavobacteriia	Flavobacteriales	Flavobacteriaceae	*Lutibacter*
0.00000	0.00257	0.00000	0.00086	0.00343	Bacteroidetes	Bacteroidia	Bacteroidales	Bacteroidaceae	*Bacteroides*
0.00000	0.00000	0.00327	0.00000	0.00327	Proteobacteria	Alphaproteobacteria	Rhodobacterales	Rhodobacteraceae	*Pseudoruegeria*
0.00000	0.00182	0.00000	0.00135	0.00318	Actinobacteria	Actinobacteria	Geodermatophilales	Geodermatophilaceae	*Modestobacter*
0.00000	0.00314	0.00000	0.00000	0.00314	Bacteroidetes	Cytophagia	Cytophagales	Cytophagaceae	*Cytophaga*
0.00000	0.00149	0.00000	0.00156	0.00306	Proteobacteria	Gammaproteobacteria	Oceanospirillales	Endozoicomonadaceae	*Kistimonas*
0.00292	0.00000	0.00000	0.00000	0.00292	Proteobacteria	Oligoflexia	Silvanigrellales	Silvanigrellaceae	*Silvanigrella*
0.00175	0.00000	0.00000	0.00112	0.00287	Proteobacteria	Alphaproteobacteria	Sphingomonadales	Sphingomonadaceae	*Zymomonas*
0.00000	0.00000	0.00281	0.00000	0.00281	Proteobacteria	Gammaproteobacteria	nd	nd	*Thiolapillus*
0.00000	0.00000	0.00271	0.00000	0.00271	Proteobacteria	Gammaproteobacteria	Cellvibrionales	Halieaceae	*Marimicrobium*
0.00000	0.00000	0.00268	0.00000	0.00268	Proteobacteria	Gammaproteobacteria	Chromatiales	Ectothiorhodospiraceae	*Inmirania*

0.00000	0.00000	0.00262	0.00000	0.00262	Proteobacteria	Gammaproteobacteria	Cellvibrionales	Halieaceae	*Halioglobus*
0.00000	0.00000	0.00255	0.00000	0.00255	Proteobacteria	Deltaproteobacteria	Desulfobacterales	Desulfobacteraceae	*Desulfococcus*
0.00000	0.00000	0.00252	0.00000	0.00252	Acidobacteria	Thermoanaerobaculia	Thermoanaerobaculales	Thermoanaerobaculaceae	*Thermoanaerobaculum*
0.00000	0.00000	0.00245	0.00000	0.00245	Proteobacteria	Alphaproteobacteria	Rhizobiales	Rhodobiaceae	*Anderseniella*
0.00000	0.00000	0.00245	0.00000	0.00245	Proteobacteria	Alphaproteobacteria	Rhodospirillales	Geminicoccaceae	*Candidatus Combothrix*
0.00000	0.00000	0.00245	0.00000	0.00245	Proteobacteria	Gammaproteobacteria	Chromatiales	Chromatiaceae	*Thiohalocapsa*
0.00000	0.00000	0.00000	0.00236	0.00236	Proteobacteria	Gammaproteobacteria	Cellvibrionales	Microbulbiferaceae	*Microbulbifer*
0.00000	0.00000	0.00229	0.00000	0.00229	Proteobacteria	Alphaproteobacteria	Rhodobacterales	Rhodobacteraceae	*Marivita*
0.00000	0.00000	0.00222	0.00000	0.00222	Proteobacteria	Deltaproteobacteria	Desulfobacterales	Desulfobacteraceae	*Desulfosarcina*
0.00000	0.00000	0.00206	0.00000	0.00206	Proteobacteria	Gammaproteobacteria	Arenicellales	Arenicellaceae	*Arenicella*
0.00000	0.00000	0.00186	0.00000	0.00186	Actinobacteria	Actinobacteria	Streptomycetales	Streptomycetaceae	*Streptomyces*
0.00000	0.00000	0.00180	0.00000	0.00180	Proteobacteria	Alphaproteobacteria	nd	nd	nd
0.00000	0.00000	0.00177	0.00000	0.00177	Proteobacteria	Alphaproteobacteria	Rhodobacterales	Rhodobacteraceae	*Sulfitobacter*
0.00093	0.00072	0.00000	0.00000	0.00164	Bacteroidetes	Bacteroidia	Marinilabiliales	Prolixibacteraceae	*Prolixibacter*
0.00000	0.00000	0.00157	0.00000	0.00157	Bacteroidetes	Flavobacteriia	Flavobacteriales	Flavobacteriaceae	*Marinirhabdus*
0.00000	0.00084	0.00072	0.00000	0.00156	Firmicutes	Bacilli	Lactobacillales	Leuconostocaceae	*Leuconostoc*
0.00154	0.00000	0.00000	0.00000	0.00154	Bacteroidetes	Cytophagia	Cytophagales	Flammeovirgaceae	*Sediminitomix*
0.00000	0.00106	0.00000	0.00047	0.00153	Firmicutes	Clostridia	Eubacteriales	Eubacteriales *incertae sedis*	*Natranaerovirga*
0.00152	0.00000	0.00000	0.00000	0.00152	Proteobacteria	Gammaproteobacteria	nd	nd	nd
0.00000	0.00000	0.00144	0.00000	0.00144	Bacteroidetes	Flavobacteriia	Flavobacteriales	Flavobacteriaceae	*Taeania*
0.00137	0.00000	0.00000	0.00000	0.00137	Proteobacteria	Gammaproteobacteria	Vibrionales	Vibrionaceae	nd
0.00000	0.00000	0.00134	0.00000	0.00134	Proteobacteria	Deltaproteobacteria	Desulfobacterales	Desulfobacteraceae	*Candidatus Magnetomorum*
0.00000	0.00129	0.00000	0.00000	0.00129	Firmicutes	Clostridia	Clostridiales	Clostridiales Family XII. Incertae Sedis	*Acidaminobacter*
0.00113	0.00000	0.00000	0.00000	0.00113	nd	nd	nd	nd	nd
0.00095	0.00000	0.00000	0.00000	0.00095	Fibrobacteres	Chitinivibrionia	Chitinivibrionales	Chitinivibrionaceae	*Chitinivibrio*
0.00000	0.00000	0.00085	0.00000	0.00085	Proteobacteria	Alphaproteobacteria	Rhodobacterales	Rhodobacteraceae	*Oceanicella*
0.00078	0.00000	0.00000	0.00000	0.00078	Bacteroidetes	Flavobacteriia	Flavobacteriales	Flavobacteriaceae	*Formosa*
0.00025	0.00048	0.00000	0.00000	0.00073	Proteobacteria	Gammaproteobacteria	Alteromonadales	Shewanellaceae	*Shewanella*
0.00071	0.00000	0.00000	0.00000	0.00071	Firmicutes	Bacilli	Bacillales	Bacillaceae	*Bacillus*
0.00000	0.00058	0.00000	0.00000	0.00058	Firmicutes	Clostridia	Clostridiales	Peptostreptococcaceae	*Paeniclostridium*
0.00000	0.00000	0.00000	0.00039	0.00039	Proteobacteria	Gammaproteobacteria	Oceanospirillales	Hahellaceae	*Zooshikella*
0.00025	0.00000	0.00000	0.00000	0.00025	Proteobacteria	Gammaproteobacteria	Alteromonadales	Colwelliaceae	*Thalassomonas*

1.2. Supplementary figures

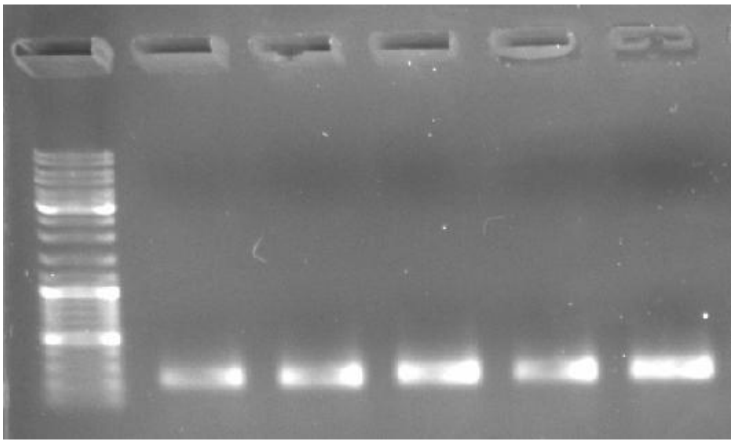

Figure S1 – Example of PCR amplifications run in an 1% agarose gel electrophoresis. The marker (1 Kb DNA ladder, ThermoScientific) is in the first lane; the next 5 lanes correspond to the amplicons of the five replicates obtained for one DNA extraction kit.

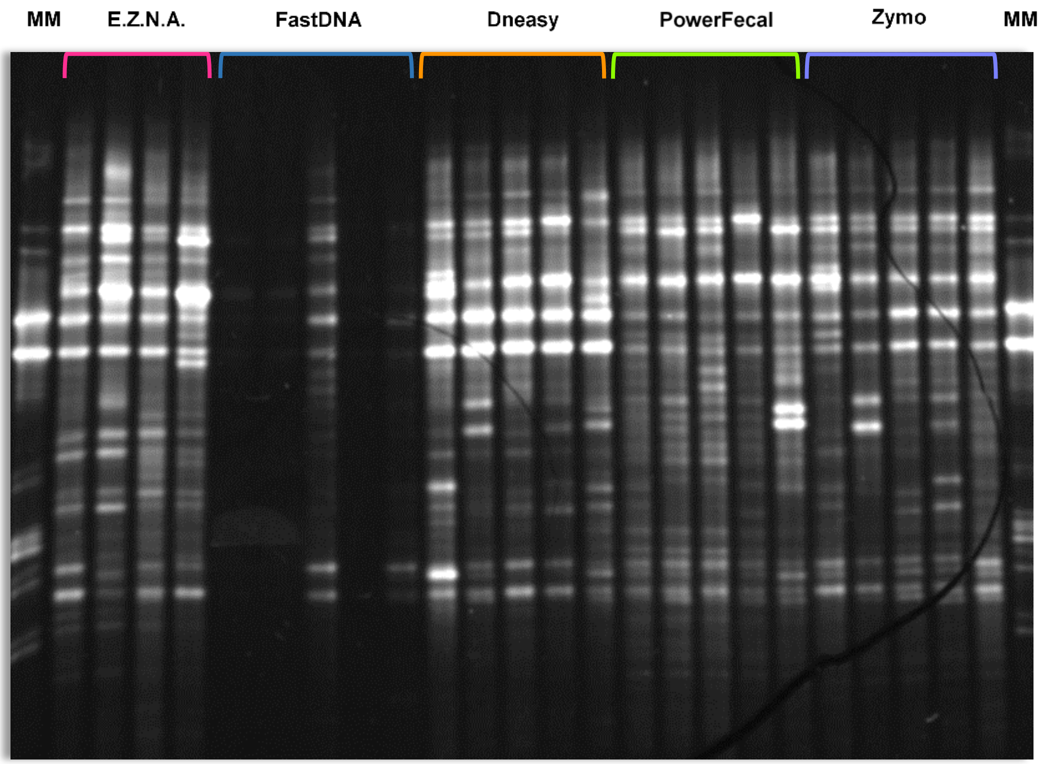

Figure S2 – DGGE profiles of the bacterial community associated with the *Cerastoderma edule* gut, using five different DNA extraction kits (indicated above the gel picture; 5 replicates were tested *per* extraction kit). MM: molecular marker.

#OTU ID	E.Z.N.A.	Dneasy	PowerFecal	Zymo	Taxonomic affiliation				
					Phylum	Class	Order	Family	Genus
OTU_10					Proteobacteria	Alphaproteobacteria	Rickettsiales	Anaplasmataceae	*Anaplasma*
OTU_3					Proteobacteria	Epsilonproteobacteria	Campylobacterales	Campylobacteraceae	*Arcobacter*
OTU_6					Proteobacteria	Gammaproteobacteria	Thiotrichales	Fastidiosibacteraceae	*Cysteiniphilum*
OTU_15					Proteobacteria	Deltaproteobacteria	Desulfobacterales	Desulfobulbaceae	*Desulfobulbus*
OTU_9					Proteobacteria	Gammaproteobacteria	Oceanospirillales	Endozoicomonadaceae	*Endozoicomonas*
OTU_11					Bacteroidetes	Bacteroidia	Marinilabiliales	Marinilabiliaceae	*Saccharicrinis*
OTU_17					Proteobacteria	Gammaproteobacteria	Chromatiales	Woeseiaceae	*Woeseia*
OTU_16					Proteobacteria	Deltaproteobacteria	Desulfobacterales	Desulfobulbaceae	*Desulfopila*
OTU_63					Proteobacteria	Gammaproteobacteria	Chromatiales	Ectothiorhodospiraceae	*Thiohalomonas*
OTU_23					Chlamydiae	Chlamydiia	Parachlamydiales	Criblamydiaceae	*Criblamydia*
OTU_28					Bacteroidetes	Flavobacteriia	Flavobacteriales	Flavobacteriaceae	*Tenacibaculum*
OTU_40					Actinobacteria	Acidimicrobiia	Acidimicrobiales	Ilumatobacteraceae	*Ilumatobacter*
OTU_34					Spirochaetes	Spirochaetia	Spirochaetales	Spirochaetaceae	*Spirochaeta*
OTU_31					Bacteroidetes	Flavobacteriia	Flavobacteriales	Flavobacteriaceae	*Lutimonas*
OTU_26					Proteobacteria	Gammaproteobacteria	Vibrionales	Vibrionaceae	*Vibrio*
OTU_49					Proteobacteria	Gammaproteobacteria	Cellvibrionales	Halieaceae	*Luminiphilus*
OTU_25					Bacteroidetes	Bacteroidia	Marinilabiliales	Marinilabiliaceae	*Saccharicrinis*
OTU_47					Bacteroidetes	Flavobacteriia	Flavobacteriales	Flavobacteriaceae	*Actibacter*
OTU_29					Proteobacteria	Gammaproteobacteria	Vibrionales	Vibrionaceae	*Vibrio*
OTU_45					Bacteroidetes	Flavobacteriia	Flavobacteriales	Flavobacteriaceae	*Actibacter*
OTU_62					Proteobacteria	Gammaproteobacteria	Chromatiales	Wenzhouxiangellaceae	*Wenzhouxiangella*
OTU_48					Proteobacteria	Gammaproteobacteria	Thiotrichales	Fastidiosibacteraceae	*Fangia*
OTU_13					Proteobacteria	Gammaproteobacteria	Vibrionales	Vibrionaceae	*Vibrio*
OTU_54					Proteobacteria	Deltaproteobacteria	Desulfobacterales	Desulfobulbaceae	*Desulfopila*
OTU_44					Proteobacteria	Epsilonproteobacteria			*Sulfurovum*
OTU_42					Proteobacteria	Gammaproteobacteria	Thiotrichales	Fastidiosibacteraceae	*Cysteiniphilum*
OTU_86					Proteobacteria	Alphaproteobacteria	Rickettsiales	Rickettsiaceae	*Rickettsia*
OTU_74					Proteobacteria	Alphaproteobacteria	Rickettsiales	Rickettsiaceae	*Rickettsia*
OTU_133					Proteobacteria	Gammaproteobacteria	Chromatiales	Ectothiorhodospiraceae	
OTU_65					Actinobacteria	Acidimicrobiia	Acidimicrobiales	Ilumatobacteraceae	*Ilumatobacter*

Legend	
	0.00%
	0.39%
	0.68%
	2.28%
	9.00%

Figure S3 – Heatmap showing the 30 predominant OTUs without *Mycoplasma* spp. in regard to the DNA extraction kit used (E.Z.N.A., DNeasy, PowerFecal, and Zymo). The colour code represents the relative abundance of OTUs (%). Values below 0.1% of relative abundance are represented in white.

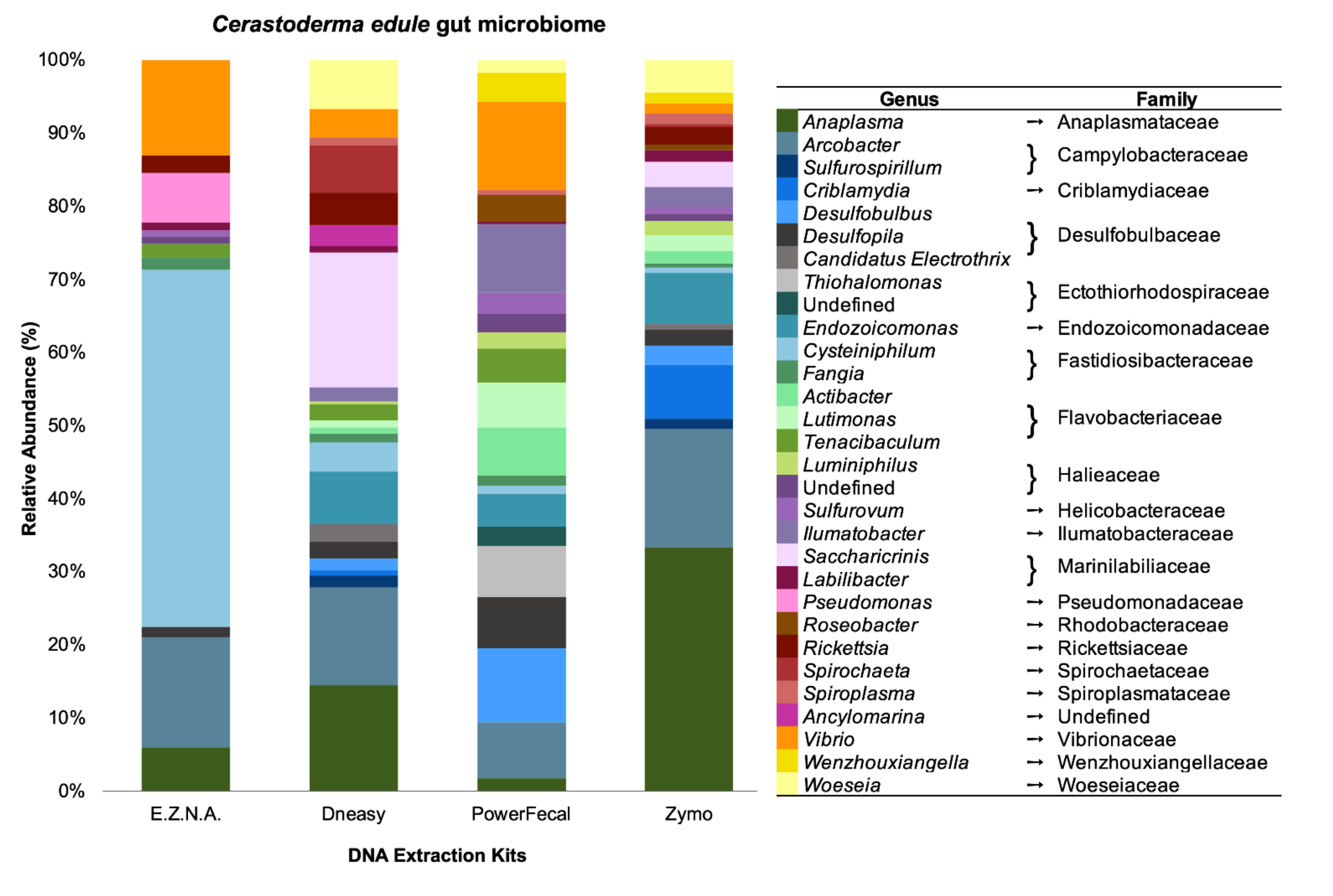

Figure S4 – Relative abundance of the 30 predominant genera without *Mycoplasma* spp. in *Cerastoderma edule* gut, following the application of four DNA extraction kits (E.Z.N.A., DNeasy, PowerFecal, and Zymo).
